# Unique structural solution from a *V*_*H*_*3-30* antibody targeting the hemagglutinin stem of influenza A viruses

**DOI:** 10.1038/s41467-020-20879-6

**Published:** 2021-01-25

**Authors:** Wayne D. Harshbarger, Derrick Deming, Gordon J. Lockbaum, Nattapol Attatippaholkun, Maliwan Kamkaew, Shurong Hou, Mohan Somasundaran, Jennifer P. Wang, Robert W. Finberg, Quan Karen Zhu, Celia A. Schiffer, Wayne A. Marasco

**Affiliations:** 1 Department of Cancer Immunology and Virology, Dana-Farber Cancer Institute, Boston, MA USA; 2 Department of Biochemistry and Molecular Pharmacology, University of Massachusetts Medical School, Worcester, MA USA; 3 Department of Medicine, University of Massachusetts Medical School, Worcester, MA USA; 4 Department of Medicine, Harvard Medical School, Boston, MA USA

**Keywords:** Influenza virus, Influenza virus, X-ray crystallography

## Abstract

Broadly neutralizing antibodies (bnAbs) targeting conserved influenza A virus (IAV) hemagglutinin (HA) epitopes can provide valuable information for accelerating universal vaccine designs. Here, we report structural details for heterosubtypic recognition of HA from circulating and emerging IAVs by the human antibody 3I14. Somatic hypermutations play a critical role in shaping the HCDR3, which alone and uniquely among *V*_*H*_*3-30* derived antibodies, forms contacts with five sub-pockets within the HA-stem hydrophobic groove. 3I14 light-chain interactions are also key for binding HA and contribute a large buried surface area spanning two HA protomers. Comparison of 3I14 to bnAbs from several defined classes provide insights to the bias selection of *V*_*H*_*3-30* antibodies and reveals that 3I14 represents a novel structural solution within the *V*_*H*_*3-30* repertoire. The structures reported here improve our understanding of cross-group heterosubtypic binding activity, providing the basis for advancing immunogen designs aimed at eliciting a broadly protective response to IAV.

## Abstract (web summary)

Previously, a broadly neutralizing antibody, 3I14, active against groups 1 and 2 influenza A viruses was isolated from human memory B cells and showed protection in mice from lethal viral challenge. Here, Harshbarger and Deming et al. provide the crystal structure of 3I14 Fab in complex with H3, H6, and H10.

## Introduction

Influenza A virus (IAV) is a persistent global health concern due to its ability to rapidly mutate and year-round efforts are required to prepare for and combat seasonal strains. Despite such effort, seasonal influenza viruses cause significant global morbidity and mortality, as well as vast social and economic burdens. In addition, the threat of a pandemic strain arising is an endless concern because of large influenza reservoirs in swine and avian populations^1^. Several avian IAVs have caused sporadic human infections in the recent past, including H7N9^2^, H5N1^3^, and H9N2^4^. The H5N1 virus infected over 800 people between 2003 and 2019 with a human fatality rate of ~53% (https://www.who.int/influenza/human_animal_interface/HAI_Risk_Assessment/en/), whereas the H7N9 virus, which was first reported in China in 2013, has over 1500 confirmed cases and ~39% human fatality rate (https://www.who.int/csr/don/05-september-2018-ah7n9-china/en/).

More recently, strains of H6N1 (A/Taiwan/2/2013) and H10N8 (A/Jiangxi-Donghu/346/2013) have caused human infections^5–8^, and although no fatalities from the H6 infections were reported, the virus does have a slight preference for human receptors and therefore may represent an intermediate towards a complete human adaptation. Conversely, H10N8 has high genetic similarity with the deadly H7N9 viruses and two of the three reported H10N8 infections were fatal. Antiviral drugs can be used to treat early stages of infection in an effort to mitigate epidemics and pandemics; however, resistance has developed in most circulating viruses to the M2 ion channel blocker adamantane^9,10^ and many viruses also have resistance to neuraminidase inhibitors^10,11^. Thus, there is an urgent need to advance efforts towards a more universal solution for protection against IAV infections.

The envelope glycoprotein hemagglutinin (HA) is the major target of the humoral immune response and is responsible for receptor attachment and entry of IAVs^12^. HA is synthesized in an inactive form (HA0) and then processed by intracellular proteases to an active state, which consists of an apical globular head domain (HA1) responsible for mediating receptor binding and a stem domain (HA2) that harbors the fusion machinery. HA1 and HA2 are linked by a single disulfide bond and assemble as trimers of heterodimers. The head domain is immunodominant^13^, but sequence diversity between IAVs and tolerance for antigenic changes make the antibodies elicited towards the head typically only viable towards highly related strains, although several receptor-binding site targeting antibodies capable of neutralizing diverse IAVs have been characterized^14,15^. The sequence diversity in IAV is exemplified by the presence of 18 HA serotypes, which can be further divided by phylogenetic relatedness into group 1 (including H1, H2, H5, H6, and H9) or group 2 (including H3, H7, and H10).

Stem-directed broadly neutralizing antibodies (bnAbs), which are elicited by natural infection and vaccination, tend to have a wider breadth than head-directed antibodies and are often capable of protecting against entire subtypes, groups, or even types^16–19^. Stem-directed bnAbs prevent the release of viral contents into the host cell by locking HA in a prefusion state, thus inhibiting the structural rearrangement necessary to fuse to the host endosomal membrane^20^. The *V*_*H*_*1-69* class of stem-directed bnAbs occurs frequently and many have been identified and structurally characterized^21,22^. With the exception of CR9114^18^ and 27F3^23^, which target both group 1 and group 2 IAVs, the majority of *V*_*H*_*1-69* antibodies are specific for group 1. Germlines *V*_*H*_*6-1*, *V*_*H*_*1-18*, and *V*_*H*_*3-30*^24–26^ have also shown bias use for the production of stem-directed bnAbs and, unlike those derived from *V*_*H*_*1-69*, both heavy and light chains are used for binding HA, with some of these characterized antibodies capable of neutralizing both group 1 and group 2 IAVs.

We recently reported on the discovery and in vivo efficacy of the *V*_*H*_*3-30*-derived bnAb 3I14^27^. MAb 3I14 was isolated from H3 (A/Brisbane/10/2007) reactive memory B cells, neutralizes group 1 and group 2 IAVs in cell culture models, and protects mice from viral challenge with lethal strains of H7N7-NL219, H7N9-AH13, H3N2-BR07-ma, and H5N1-VN04 viruses^27^. Similar to other stem-directed bnAbs, 3I14 prevents proteolytic cleavage of HA0, inhibits a pH-induced conformational change in HA and promotes antibody-dependent cellular cytotoxicity. A point mutation in the light variable domain, termed 3I14^D93N^ (3I14^D94N^ with prior numbering), was found to increase binding affinity and viral neutralization potential by approximately tenfold against H5, with no negative effect on binding or neutralization to H3.

In this work, we elucidate the structural determents for cross-group binding of HA by 3I14 through solving the crystal structures of the 3I14 fragment antigen-binding region (Fab) complexed with HA from group 1 (H6) and group 2 (H3 and H10). We find that 3I14 uses a unique binding mechanism for recognition of the HA stem, whereby only HCDR3 residues make contacts within the hydrophobic groove, whereas the light chain provides a large footprint spanning two HA protomers. 3I14 is highly mutated and the location of these mutations in the heavy and light chains mold the HCDR3 into the shape needed for recognizing the HA stem. Structures of 3I14^D93N^ bound to HA from group 1 and group 2 reveal the molecular basis for enhanced binding affinity towards H6. In addition, comparison of 3I14 with other *V*_*H*_*3-30*-derived bnAbs, as well as bnAbs from diverse germlines, reveals that 3I14 represents a unique example of a stem-directed bnAb. This work broadens our understanding of HA stem recognition by bnAbs and the structural diversity of bnAbs that can be generated by the *V*_*H*_*3-30* scaffold.

## Results

### Binding of 3I14 to human infecting H6 and H10 HAs

The recent emergence into humans of avian H10N8^7^ (A/Jiangxi-Donghu/346/2013) (group 2) and H6N1^8^ (A/Taiwan/2/2013) (group 1) infections led us to investigate whether mAbs 3I14 and 3I14^D93N^ could bind HA from each virus with similar binding affinities as we observed with other HA strains^27^. Neither of these HAs were included in our original characterization of 3I14, although phylogenetically similar viruses to H6 (such as H1 and H5) and H10 (such as H7, and H9) were studied^27^. This strain of H6 has residue Glu39_HA2_, which in H5 led to the engineering of 3I14^D93N^ and enhanced binding affinity; therefore, we hypothesized that 3I14^D93N^ may also bind H6 with improved affinity. 3I14 and 3I14^D93N^ bound H10 with a similar equilibrium binding constant (*K*_D_) of ~0.4 nM (Table 1 and Supplementary Fig. 1). In contrast and as predicted, the wild-type 3I14 lost approximately sixfold in the *K*_D_ to H6, whereas 3I14^D93N^ maintained this potent binding affinity to H6 (~3 nM for wild-type 3I14 and 0.4 nM for 3I14^D93N^) (Table 1 and Supplementary Fig. 1).

**Table 1:** Binding data for 3I14 and 3I14^D93N^.

3I14 Variant	H6 (A/Taiwan/2/2013)			H10 (A/Jiangxi-Donghu/346/2013)			H3 (A/Perth/16/2009)^a^		
	*K*_a_ (M^−1^ s^−1^)	*K*_d_ (s^−1^)	*K*_D_ (nM)	*K*_a_ (M^−1^ s^−1^)	*K*_d_ (s^−1^)	*K*_D_ (nM)	*K*_a_ (M^−1^ s^−1^)	*K*_d_ (s^−1^)	*K*_D_ (nM)
3I14 WT	5.19 × 10^4^	1.52 × 10^−4^	2.9	1.13 × 10^5^	5.38 × 10^−6^	0.48	1.52 × 10^5^	3.99 × 10^−5^	0.26
3I14^D93N^	6.90 × 10^4^	3.08 × 10^−5^	0.45	1.23 × 10^5^	5.00 × 10^−6^	0.41	1.77 × 10^5^	5.44 × 10^−5^	0.31

^a^Reported by Fu et al.^27^.

To determine whether the improved binding affinity of the 3I14^D93N^ mutant was a result of stabilization of the 3I14 IgG, we measured the melting temperatures (*T*_M_) for wild-type 3I14 and 3I14^D93N^ IgG. We found that the *T*_M_ was 78 °C in each case (Supplementary Fig. 2), signifying that the light-chain Asp93Asn mutation does not impact the IgG thermostability. Rather, a reduction in the antibody off-rate by approximately fivefold, with little change in the on-rate, suggests a direct role for this residue in binding HA.

### Structure of 3I14 Fab in complex with group 1 and 2 HAs

To investigate the structural basis for heterosubtypic recognition of IAV by 3I14, as well as confirm the molecular details for the enhanced binding affinity of 3I14^D93N^ to H6, crystal structures for the 3I14 and 3I14^D93N^ Fabs in complex with HAs from group 1, H6 (A/Taiwan/2/13), and group 2, H3 (A/Victoria/361/2011) and H10 (A/Jiangxi/IPB13/2013) were solved. A total of four complex structures permitted a thorough understanding of the 3I14- and 3I14^D93N^-binding modes: H3:3I14, H3:3I14^D93N^, H6:3I14^D93N^, and H10:3I14 at resolutions of 3.5 Å, 3.2 Å, 3.5 Å, and 4.2 Å, respectively (Table 2). The structures of the HA proteins in each complex are similar to those of the apo structures, which have been previously reported (Supplementary Fig. 3)^28,29^. The electron densities for the H3 and H6 complexes are clear and well-ordered at the HA:Fab interfaces, allowing for the complementarity determining regions (CDRs) of 3I14 to be manually built into the H3 bound structure (Supplementary Fig. 4). The H10 structure has clear density for the C-α-backbone for both the HA and Fab portions; however, side-chain residues are not well resolved and some density is missing near the fusion peptide. Therefore, our description of the intermolecular contacts is based only on the H3 and H6 structures.

**Table 2:** X-ray data collection and crystallographic refinement statistics.

	H3_3I14	H3_D93N	H6_D93N	H10_3I14
PDB ID	6WF0	6WEZ	6WEX	6WF1
Data collection				
Space group	*P*6_3_	*P*6_3_	*R*32	*P*321
Cell dimensions				
*a* (Å)	130.32	130.84	117.81	127.02
*b* (Å)	130.32	130.84	117.81	127.02
*c* (Å)	188.55	189.34	438.26	158.37
*α*, *β*, *γ* (°)	90, 90, 120	90, 90, 120	90, 90, 120	90, 90, 120
Resolution (Å)	42.66–3.46 (3.58–3.45)	39.02–3.21 (3.32–3.21)	48.26–3.49 (3.60–3.49)	41.58–4.19 (4.34–4.19)
Unique reflections	23,612 (2349)	29,818 (2918)	15,294 (1421)	9561 (775)
Total reflections	130,821 (12,953)	140,225 (14,027)	144,741 (13,615)	32,231 (1783)
*R*_merge_	0.196 (0.992)	0.141 (1.147)	0.168 (0.649)	0.110 (0.581)
*R*_pim_	0.090 (0.459)	0.072 (0.581)	0.058 (0.219)	0.063 (0.406)
*I*/*σI*	8.9 (1.7)	10.2 (1.2)	9.9 (3.2)	8.8 (1.5)
CC_1/2_	0.989 (0.390)	0.996 (0.596)	0.995 (0.86)	0.987 (0.646)
Completeness (%)	99.1 (98.7)	98.8 (97.6)	99.3 (94.5)	84.5 (70.2)
Redundancy	5.5 (5.5)	4.7 (4.8)	9.5 (9.6)	3.4 (2.3)
Refinement				
Resolution (Å)	42.66–3.46	39.02–3.21	48.26–3.49	41.58–4.19
Unique reflections	23,516 (2353)	29,741 (2964)	15,291 (1421)	9564 (778)
*R*_work_/*R*_free_	0.231/0.282	0.232/0.281	0.276/0.333	0.261/0.309
No. atoms:				
Protein	6959	6959	7109	6959
Ligand	211	206	14	14
B-factors (Å^2^)				
Average	100	95	112	244
Hemagglutinin	79	73	86	235
Glycans	121	111	108	257
Antibody	124	120	145	256
R.m.s. deviations				
Bond lengths (Å)	0.003	0.003	0.002	0.002
Bond angles (°)	0.66	0.68	0.63	0.49
Ramachandran				
Favored (%)	92.16	94.92	92.31	94.51
Allowed (%)	7.84	5.08	7.58	5.49
Outliers (%)	0	0	0.11	0

*R.m.s. deviations* root-mean-square deviations.

*Values in parentheses are for highest-resolution shell.

**Fig. 1:**
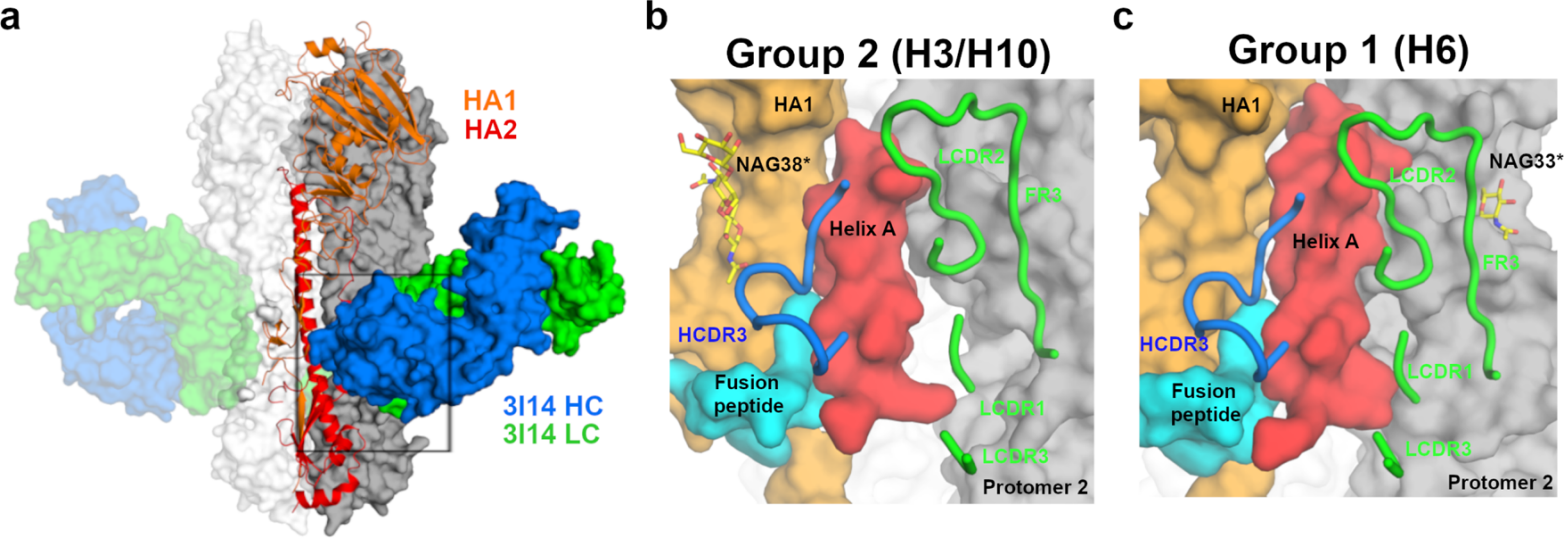
3I14 recognition of group 1 and group 2 HA proteins. **a** X-ray structure of H3 hemagglutinin bound by Fab 3I14. One HA protomer is shown in cartoon with HA1 colored orange and HA2 colored red. The other two HA protomers are shown in surface representation and colored white or gray. 3I14 is shown in surface representation with heavy chain colored blue and light chain colored green. **b** Zoomed in view of the box from **a** showing the H3-3I14 interface and the major structural elements of HA, which are recognized. Helix A is colored red, the fusion peptide is colored cyan, HA1 is colored orange, and a second HA protomer is colored gray. The 3I14 CDRs are shown as cartoons. A group 2-specific sugar residue at position 38 of HA1 is labeled as NAG38*. **c** Similar view as in **b** showing the H6-3I14^D93N^ interface. A group 1-specific sugar on the second HA protomer, which interacts with 3I14 is labeled as NAG33*.

3I14 recognizes the stem of group 1 and 2 HAs in a manner consistent with findings that it prevents proteolytic cleavage of the HA precursor^27^ and in an overlapping region as other reported stem-directed bnAbs^22,24–26,30–32^ (Fig. 1a and Supplementary Fig. 5). The 3I14 epitope spans to a second HA protomer, which results in a total buried interface area on HA of 980 Å^2^, consistent with the  high binding affinity (Fig. 1b, c). Heavy-chain interactions occur solely through contacts with HCDR3, which buries a surface area of 481 Å^2^ in the hydrophobic groove, between the middle and lower portions of Helix A, the fusion peptide, and HA1.

**Fig. 2:**
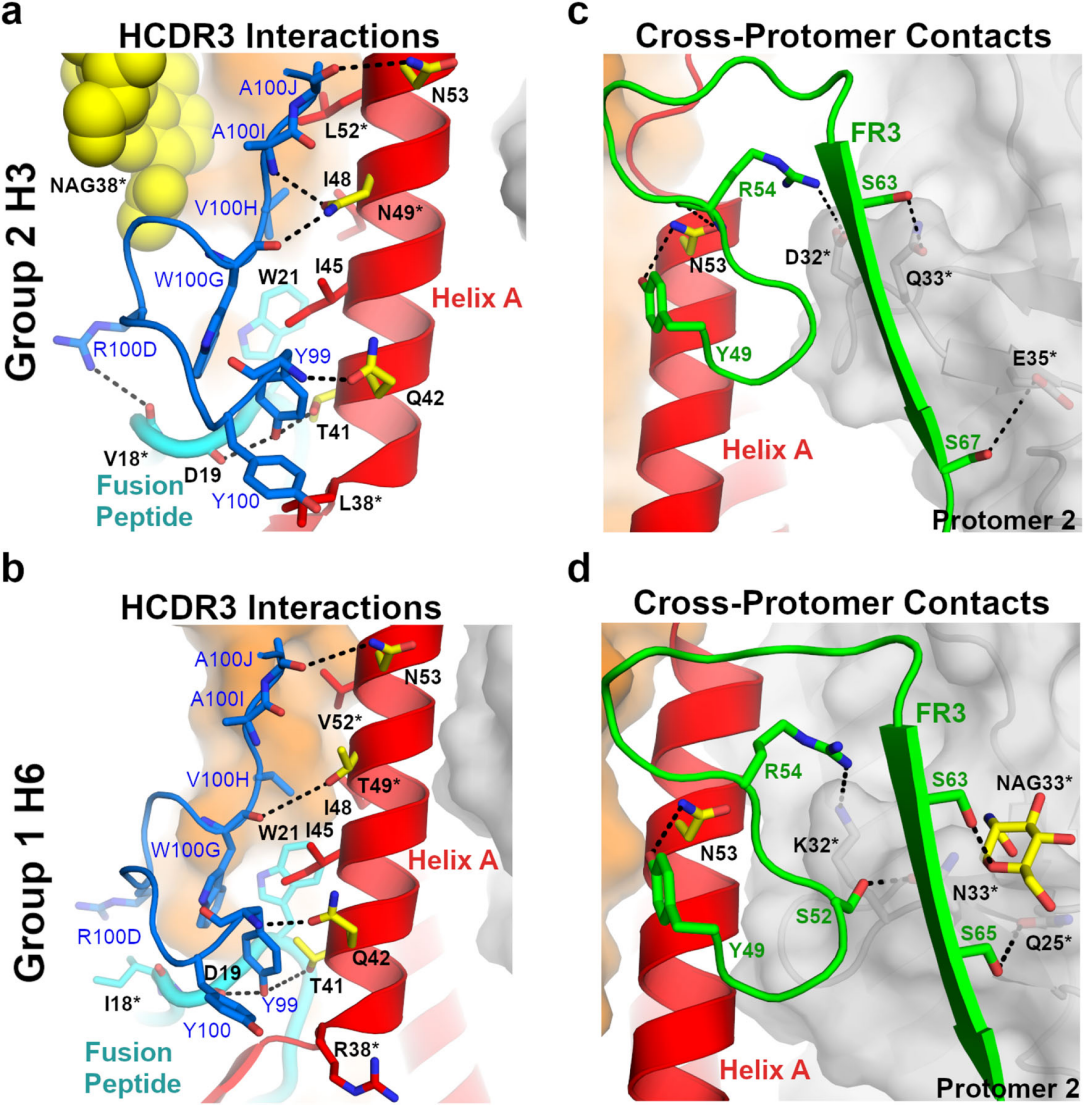
Binding comparison of 3I14 and 3I14^D93N^ with group 2 and group 1 HAs. **a**, **b** 3I14 HCDR3 interactions with either group 2 H3 (**a**) or group 1 H6 (**b**) HAs. The 3I14 HCDR3 is colored blue with residues that make either polar or vdWs interactions shown as sticks and labeled in blue. The HA helix A is shown in cartoon and colored red, the fusion peptide is colored cyan, HA1 is colored orange, and the group 2-specific glycan is shown as yellow spheres. Helix A residues that make polar interactions are shown as yellow sticks, whereas vdWs contacts are shown as red sticks. Dashed lines indicate hydrogen bonds. Residues that differ between group 1 and group 2 HAs are indicated with an asterisk. **c**, **d** 3I14 LCDR2 and FR3 interactions, highlighting cross-protomer contacts. 3I14 light chain is colored green and shown as cartoon with green labels. HA coloring and labels follow that of (**a**, **b**), except the group 1-specific glycan is shown as yellow sticks. Protomer 2, which is buried by FR3, is colored gray.

The HCDR3 is 23 residues in length, with 5 residues (Tyr99, Tyr100, Trp100G, Val100H, and Ala100J) (Kabat numbering) making extensive van der Waals (vdW) and hydrogen-bonding interactions (Fig. 2a, b). The reliance on HCDR3 for recognizing the hydrophobic groove is in contrast to other stem-directed bnAbs, which use a combination of heavy-chain or heavy- and light-chain CDRs for interacting with similar portions on HA^18,22–24^. The HCDR3 forms a six-residue loop extending away from the hydrophobic groove consisting of residues Phe100A through Val100F. At the center of the loop, Arg100D protrudes along the fusion peptide and can make polar interactions with the main-chain oxygen for either Val18 (group 2) or Ile18 (group 1). A contributing factor to the wide neutralization breadth of 3I14 is the ability to accommodate amino acid differences between group 1 and 2 HAs (Fig. 2a–d). For example, Tyr100_HCDR3_ makes vdWs interactions with Leu38_HA2_ (group 2) or the aliphatic side chain of Arg38_HA2_ (group 1) and Trp100G_HCDR3_ forms vdW contacts with Trp21_HA2_ and Ile45_HA2_ on helix A, as well as a hydrogen bond between the main-chain oxygen with either Asn49_HA2_ (group 2) or Thr49_HA2_ (group 1). Val100H makes vdW contacts with Ile48_HA2_ and Trp21_HA2_, whereas Ala100J makes vdW contacts with Leu52_HA2_ (group 2) or Val52_HA2_ (group 1). The main-chain oxygens for Ala100J_HCDR3_ and Ser97_HCDR3_ each make hydrogen bonds with the conserved residues Asn53 and Gln42, respectively. Another contributing factor to effectively neutralizing group 1 and group 2 viruses is the local flexibility of HCDR3 at Trp100G, which maintains an ~3–4 Å distance from the group-specific orientation of Trp21_HA_ (Supplementary Fig. 6). Collectively, the HCDR3 accounts for six hydrogen-bonding interactions and similar vdW contacts in each complex.

The 3I14 light chain plays a prominent role in binding and buries over half of the total surface area on HA (499 Å^2^), making contacts through LCDR2, LCDR3, and LFR3. In particular, LFR3 is responsible for nearly half the light chain buried surface area by mediating contacts with the adjacent protomer (Fig. 2c, d). This large buried surface area of the light chain is uncommon, as typically the heavy chain contributes more to binding than the light-chain^33^. The 3I14 light chain is also able to accommodate amino acid substitutions between group 1 and 2 HAs, such as Arg54_LCDR2_, which forms a cross-protomer hydrogen bond with the side chain of either Asp32_HA1_ (group 2) or Lys32_HA1_ (group 1). In addition, Ser63_LFR3_ hydrogen bonds with the side chain of Gln33_HA1_ (group 2) or Asn33_HA1_ (group 1) on protomer 2 and Ser67_LFR3_ hydrogen bonds with the side chain of Glu35_HA1_ (group 2), whereas Ser65_LFR3_ can hydrogen bond with Gln25_HA1_ (group 1). Despite the long span of the 3I14 epitope, the serine residues along LFR3 are able to accommodate the group 1 glycan located on Asn33_HA1_ of the second HA protomer, whereas more bulky residues on LFR3 would likely result in a steric clash, preventing such extensive cross-protomer interactions, and possibly limiting the ability of 3I14 to bind.

### Role of somatic mutations in shaping HCDR3

**Fig. 3:**
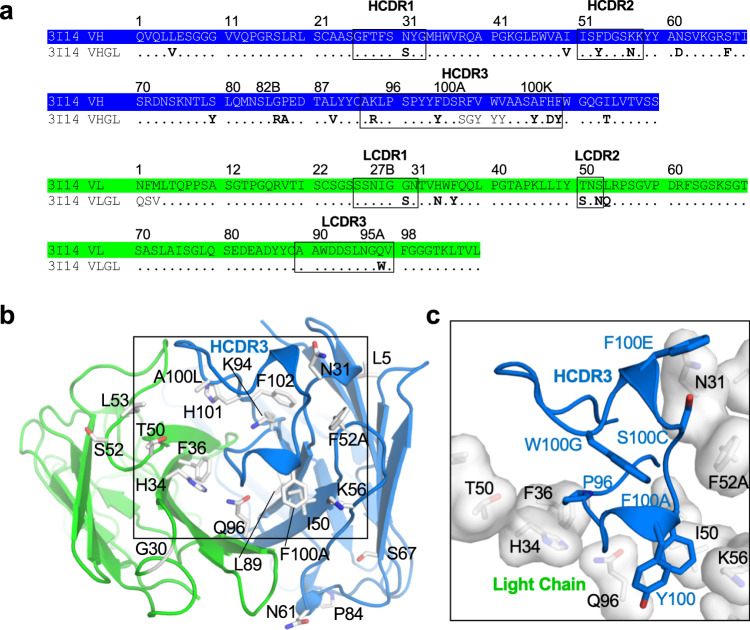
3I14 Somatic hypermutations shape HCDR3. **a** Sequences for 3I14 heavy- and light-chain wild type and germline. Somatic hypermutations (SHMs) are in bold. **b** SHMs on the 3I14 heavy and light chains are shown as white sticks and labeled. **c** Zoomed in box from **b** showing the SHMs that surround the HCDR3 as white transparent surfaces and sticks. HCDR3 is shown as a cartoon and sticks. The HCDR3 is shaped by interactions with the SHMs, which surround it.

3I14 is encoded by the IGHV3-30*18 and IGLV1-44*01 germline genes. The HCDR3 uses the IGHD3-22*01 DH segment flanked by large N-additions at both VH and IGHJ4*02 junctions^27^. We previously reported that germline reversions of somatic hypermutations (SHMs) for either the heavy chain (13 SHMs) or light chain (7 SHMs) resulted in a 2- to 7-fold reduction in binding affinity to H3 and H5, whereas simultaneous reversion of both chains to germline decreased binding affinities by approximately 14-fold in either case^27^. Six of the seven light-chain SHMs are in LCDR2 and LFR2, and mapping them on the structure showed that they cluster at the interface with the heavy chain (Fig. 3a, b). The exception is Gly30_LCDR1_, which is mutated from a serine in the germline and appears to prevent either a steric clash or the polar serine side chain from pointing towards the aliphatic side chain of Lys39_HA2_ (group 2) or Glu39_HA2_ (group 1) (Supplementary Fig. 7). Due to the location for majority of these SHMs, one possible consequence is the stabilization of the 3I14 heavy–light chain interface, which has been shown to be a critical feature in antibody affinity maturation^34,35^.

Heavy-chain SHMs are spread across HCDR1, HCDR2, FR3, and HCDR3 (Fig. 3a, b). Although HCDR1 and HCDR2 do not directly interact with HA, the SHMs occur at positions that seemingly “mold” the HCDR3 into the conformation needed to recognize the large area within the hydrophobic groove (Fig. 3c). When viewed along with the light-chain SHMs, the HCDR3 is sandwiched between seven total mutations, including a pocket formed by the light-chain somatically mutated residues His34_LFR2_ and Phe36_LFR2_, which may serve to anchor Pro96_HCDR3_. The large decrease in binding affinity obtained with either heavy- or light-chain germline-reverted mutants could therefore be due to plasticity of the HCDR3 that may result from the lack of some, or all, of these interactions.

### Molecular basis for improved binding affinity of 3I14^D93N^ to H6

**Fig. 4:**
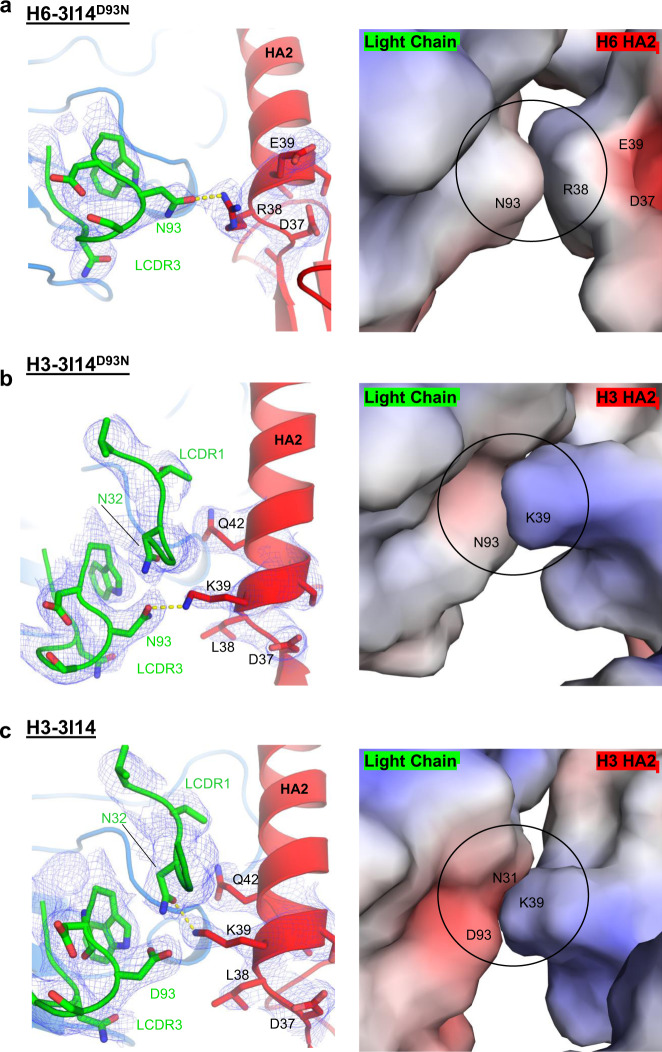
3I14 and 3I14^D93N^ binding to H6 and H3 HAs. **a** H6-3I14^D93N^ complex; **b** H3-3I14^D93N^ complex; **c** H3-3I14 wild-type complex. Left panels show the LCDR3 as green cartoon and sticks, and HA shown as red cartoon and sticks. 1*σ* 2*F*_o_–*F*_c_ electron densities are shown in blue. Hydrogen bonds are depicted as yellow dashed lines. Panel on the right is an electrostatic surface representation of the image from the left.

The 3I14^D93N^ mutant shows increased binding affinity towards recombinant H6, while retaining similar binding affinity towards H3 and H10, which was anticipated based on our previous studies^27^. In the H6-bound structure, the electron density around Asn93_LCDR3_ is well resolved and shows a hydrogen bond being formed with Arg38_HA2_ (Fig. 4a). The paratope–epitope surface potentials are each relatively neutral, with the local environment of 3I14^D93N^ being slightly negative despite the positively charged Asn93 substitution (Fig. 4a). In addition, the residues on HA in close proximity to Arg38_HA2_, namely Glu39 and Asp37, diminish the positive charge that Arg38 would carry, thus facilitating better charge complementarity with 3I14^D93N^ and explaining the improved binding characteristics.

In the H3-3I14^D93N^ structure, the Asn93_LCDR3_ forms a hydrogen bond with Lys39_HA_, and consistent with the H6-3I14^D93N^ structure, reveals a negatively charged contact surface for LCDR3, whereas in this case the H3 surface is positively charged (Fig. 4b). In the H3-3I14 wild-type structure, Asp93_LCDR3_ does not hydrogen bond with Lys39_HA_, evident by the clear electron density around each side chain (Fig. 4c). Rather, Lys39_HA_ assumes an alternate rotamer with the side chain extending towards the 3I14 LCDR1 and forming a hydrogen bond with Asn32_LCDR1_. This orientation extends the Lys39_HA_ side chain towards the center of the negative patch formed by Asp93_LCDR3_ and Asn31_LCDR1_. The ability for Lys39_HA_ to adopt multiple rotamers and for 3I14 and 3I14^D93N^ to retain a hydrogen bond interaction in either instance explains the similar binding affinities for each antibody towards group 2 HAs.

### Comparison of 3I14 binding with other V_H_3-30 derived bnAbs

**Fig. 5:**
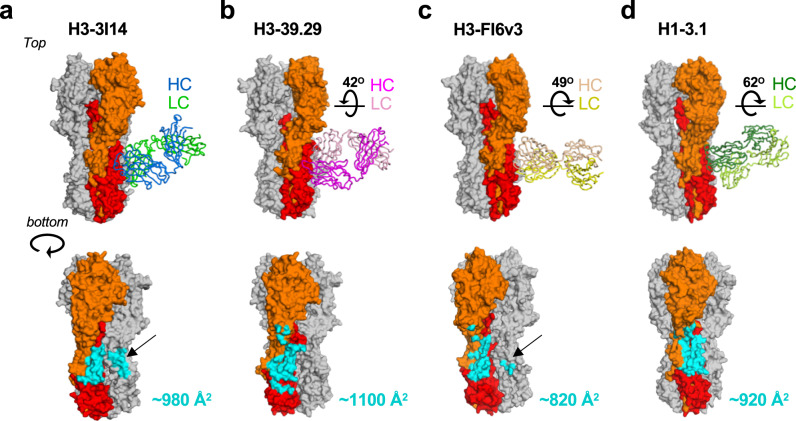
Angles of approach towards HA for *V*_*H*_*3-30* BnAbs. **a**–**d** Top: angles of approach for each of the *V*_*H*_*3-30* antibodies (H3-39.29, PDB 4KVN; H3-FI6v3, PDB 3ZTJ; H1-3.1, PDB 4PY8). HA is shown in surface representation with HA1 and HA2 from the primary protomer colored orange and red, respectively. Each Fab is shown in cartoon representation with 3I14 colored as in Fig. 4, and 39.29, FI6v3, and 3.1 colored according to the labels. The degrees shown in figures **b**–**d** is the rotation necessary to superimpose the V_H_ domain of that antibody onto the V_H_ domain of 3I14, as determined in Coot. Bottom: HA portion for each complex from the top panel, rotated ~45^o^. The buried surface area for each antibody is colored cyan on HA. The black arrows in **a** and **c** indicate cross-protomer interactions.

3I14 is the fourth bnAb to be structurally characterized which utilizes the *V*_*H*_*3-30* germline gene, along with mAbs 3.1, 39.39, and FI6v3 (stabilized version of FI6)^24–26^. MAb 3.1, which was selected by phage display shows heterosubtypic neutralization against the H1a clade (H1, H2, H5, H6) and some H9 subtypes, whereas 39.39 and FI6v3 each neutralize group 1 and 2 viruses. To better understand how the developmental pathways for each of these *V*_*H*_*3-30* bnAbs leads to broad HA recognition, the HCDR3 length, the percentage of residues in the heavy and light chains that undergo SHM, and the paratope buried surface areas were compared. This analysis revealed few distinguishing features other than 3I14, FI6v3, and 39.29 each contributing ~200–600 Å^2^ of paratope light-chain buried surface area (Supplementary Fig. 8). However, the angle of approach for each antibody towards the HA stem differs. With respect to 3I14, the V_H_ domains for 39.29, FI6v3, and 3.1 are rotated by ~42^o^, 49^o^, and 62^o^, respectively, with FI6v3 and 3.1 each rotating counterclockwise, and 39.29 rotating clockwise (Fig. 5a–d, top). In addition, FI6v3 approaches HA in a perpendicular manner, whereas each of the other antibodies is angled ~20–45^o^ towards the HA head domain. The angles of approach result in distinct but overlapping footprints on the HA stem, covering regions of HA1 and HA2. MAb 39.29 buries ~1100 Å^2^ on HA, whereas FI6v3 buries ~820 Å^2^ and 3.1 buries ~920 Å^2^ (Fig. 5a–d, bottom). The angle taken by mAb 3.1 results in the HA buried surface being nearly evenly split between HA1 and HA2; however, a potential clash with the conserved group 2 glycan at Asn32 likely contributes to group 1 selectivity. FI6v3 makes contacts with an adjacent protomer, albeit to a lesser extent than seen with 3I14 (230 Å^2^ for 3I14 vs. 90 Å^2^ for FI6).

**Fig. 6:**
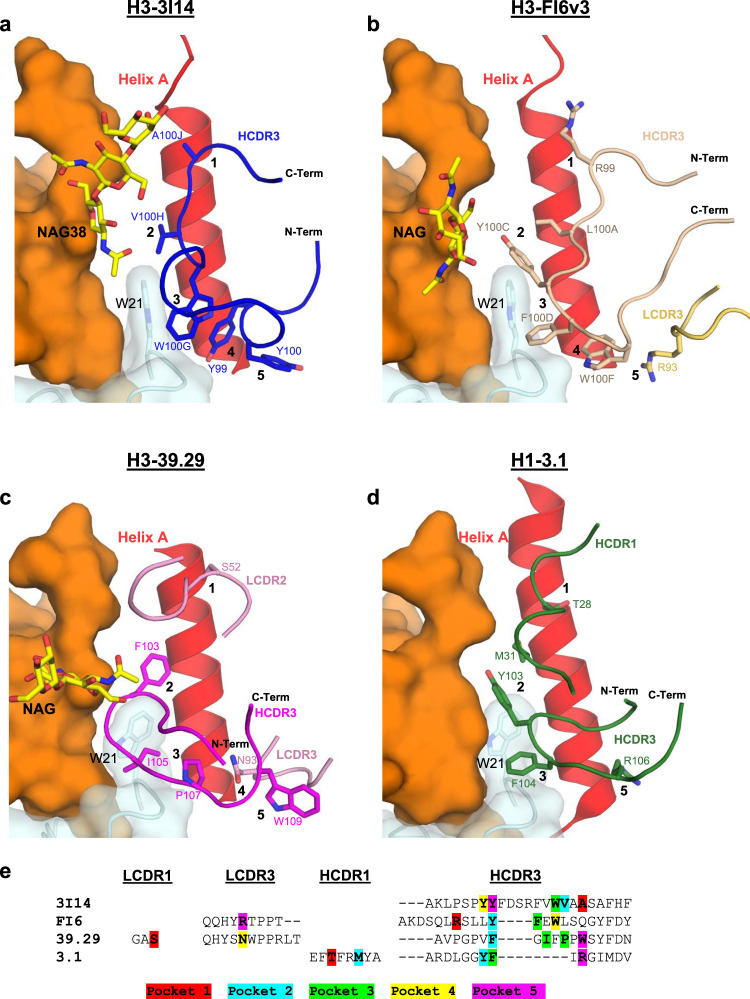
Structural comparison of *V*_*H*_*3-30*-derived bnAbs bound to HA. **a** H3-3I14; **b** H3-FI6v3; **c** H3-39.29; **d** H1-3.1. All *V*_*H*_*3-30*-derived antibodies recognize the hydrophobic groove between helix A and the fusion peptide. CDRs are shown as cartoons with residues interacting with sub-pockets (labeled as 1 through 5) shown as sticks. The fusion peptide is colored cyan with Trp21 shown as sticks; helix A is colored red and HA1 is colored orange. **e** Sequences for antibody CDRs, which have at least one residue that interacts with a sub-pocket. Highlighted residues coincide with which HA subpocket the interaction occurs: pocket 1-red; pocket 2-cyan; pocket 3-green; pocket 4-yellow; and pocket 5-purple.

Although approaching from different angles, each antibody does make extensive contacts within the HA hydrophobic groove. These interactions can be summarized by contacts with five sub-pockets, numbered as one through five starting from the top of helix A, distal from the membrane proximal region (Fig. 6a–d). Of note, each antibody shares the same V_H_–J_H_ gene segment usage (*V*_*H*_*3-30*, *J*_*H*_*4*)^24–26^ and variable D_H_ segments, which results in HCDR3s that are utilized for binding pockets two and three, centering around contact with Trp21_HA_ (Fig. 6e). Interactions with the remaining pockets are not conserved and take a variety of forms. For instance, 3I14 exclusively utilizes HCDR3 to interact with each of the five pockets, whereas FI6v3 and 39.39 incorporate light-chain CDRs and 3.1 engages HCDR1.

Another distinguishing feature for 3I14 is that the HCDR3 is flipped ~180^o^ compared to the other *V*_*H*_*3-30* antibodies, a reflection of the different approach angles (Fig. 6a). Although FI6v3 most closely resembles 3I14 by predominately utilizing HCDR3 (residues Arg99, Leu100A, Phe100D, and Trp100F) for pockets one through four, interaction with pocket five requires recruitment of the somatically mutated residue Arg93_LCDR1_ (Fig. 6b). An Arg93Ser germline-reverted mutant was shown to have a decrease in binding affinity to group 2 HAs by a factor of 44, thus demonstrating the importance for this specific interaction in the maturation of FI6^25^.

### Comparison with stem-directed antibodies from other germlines

**Fig. 7:**
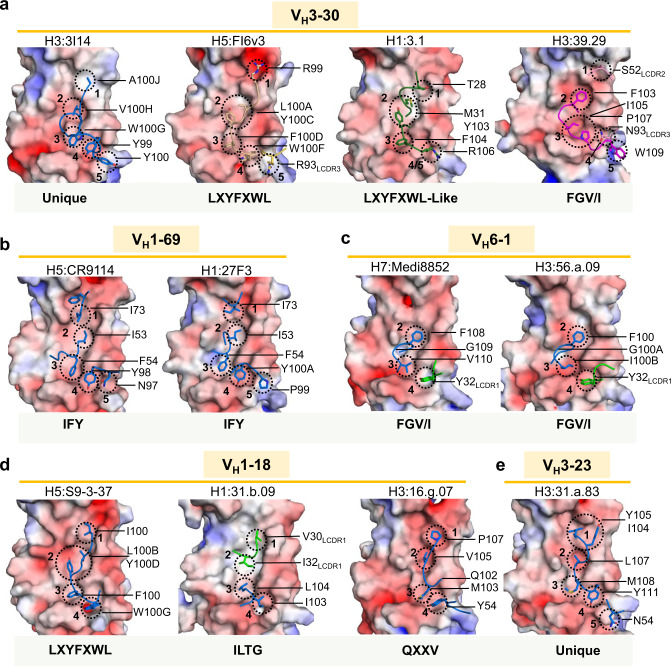
Comparison of the 3I14 HA stem hydrophobic groove interactions with other stem-bound antibodies. **a** *V*_*H*_*3-30* interactions with the hydrophobic groove are shown with heavy and light-chain CDRs colored as in Fig. 6. The HA stem is shown in surface representation and colored by electrostatic charge (red-negative, blue-positive, white-neutral). Residues interacting with pockets 1–5 are circled with a dotted line and labeled. **b** Antibodies CR9114 (PDB 4FQI) and 27F3 (PDB 5WKO) from the *V*_*H*_*1-69* germline are shown. **c** Antibodies Medi8852 (PDB 5JW4) and 56.a.09 (PDB 5K9K) from the *V*_*H*_*6-1* germline. **d** *V*_*H*_*1-18* antibodies 31.b.09 (PDB 5K9O), S9-3-37 (PDB 6E3H), and 16.g.07 (PDB 5KAN). **e** The *V*_*H*_*3-3* antibody 31.a.83 (PDB 5KAQ). For **b**–**e**, the heavy-chain CDRs are colored blue and the light-chain CDRs are colored green. The molecular signature is indicated below each figure or indicated as unique if no signature has been identified.

Next, the hydrophobic groove interactions between the *V*_*H*_*3-30*-derived antibodies with antibodies from germlines that fall into specific antibody classes were compared (Fig. 7a–d). *V*_*H*_*1-69*-derived antibodies, such as CR9114^18^ and 27F3^23^, contain an IFY motif consisting of an HCDR3 tyrosine (Y) located at position 98 or 99 coupled with HCDR2 residues Ile54 (I) and Phe55 (F) (Fig. 7b). As with all the stem-directed antibodies, which have been characterized from this class, CR9114 and 27F3 utilize HFR3 and HCDR2 for majority of the contacts within the hydrophobic groove and do not share extensive molecular similarities with any of the *V*_*H*_*3-30* antibodies. The only similarity with 3I14 is the HCDR3 tyrosine used for binding pocket four.

The *V*_*H*_*6-1*- and *V*_*H*_*1-18*-derived antibodies, on the other hand, utilize molecular signatures, which are shared by 39.29 and FI6v3, respectively. Medi8852 and 56.a.09 are representative of the *V*_*H*_*6-1* + *HD3-3* class of antibodies that use the same FGV/I motif as 39.29, where an *HD3-3* encoded phenylalanine recognizes pocket 2, and either a germline-encoded valine or the SHM-altered isoleucine binds pocket 3 (Fig. 7c). The *V*_*H*_*1-18* germline contains multiple classes, including the *HD3-9* class such as antibody S9-3-37, which harbors an LXYFXWL motif^22^ (Fig. 7d). FI6 falls into this class, which results in placement of Tyr and Phe into pockets two and three, respectively. Mab 3.1, although not strictly harboring this motif, does share the Tyr and Phe interactions. Antibody 31.b.09 (Fig. 7d) also utilizes the *HD3-9* gene (translated in a different reading frame than S9-3-37 and FI6) and harbors an ILTG motif, which places Leu in pocket three rather than a Phe. Mab 16.g.07 (*V*_*H*_*1-18*, *D2-15*, *J*_*H*_*2*) is of a multi-donor class that shares a SHM-derived Thr52_HCDR2_ and an HCDR3 QXXV motif^22^ (Fig. 7d). The binding for 16.g.07 appears most similar to mAb 31.a.83 (*V*_*H*_*3-23*, *D3-9*, *J*_*H*_*6*) (Fig. 7e), which is currently characterized as a unique lineage^22^ and binds the hydrophobic groove primarily through HCDR3 (Fig. 7e).

In summary, this comprehensive comparison reveals that the V(D)J arrangement for the *V*_*H*_*3-30* antibodies can develop multiple signatures to target the HA stem. In some cases, these HCDR3 signatures converge on similar structural solutions that have been utilized by other V_H_ germline segments. 3I14 is exclusive in the ability to solve this landscape problem by reverse orientation of the HCDR3 and in doing so makes contact with all five hydrophobic pockets. In addition, the 3I14 light chain promotes cross-protomer binding and proper positioning of the HCDR3 into the stem groove. At present, 3I14 falls into an uncharacterized class that represents a unique example among the *V*_*H*_*3-30*-derived bnAbs.

## Discussion

Broadly neutralizing human mAbs targeting the stem region of influenza HA provide opportunities for antibody-based therapeutics against yearly circulating IAV strains and treatment for novel emerging HA subtypes from zoonotic sources. Structural information obtained from antibody-HA complexes may also guide the rational design of a sought-after universal vaccine, which could protect across strains within an influenza subtype, across subtypes within IAV groups, or across all influenza A and B viruses.

In this study, we elucidated the structural basis for heterosubtypic recognition of HA by the *V*_*H*_*3-30*-derived mAb 3I14, which was originally isolated from human memory B cells^27^. Structures of 3I14 in complex with group 1 H6 as well as group 2 H3 and H10 yielded the expected result in that 3I14 recognizes HAs from each strain in a highly similar manner, consistent with other antibodies targeting stem and non-stem epitopes that have been reported elsewhere^22,25,30,36,37^. The 3I14 heavy-chain interactions consist solely of HCDR3 contacts within five sub-pockets of the stem hydrophobic groove, whereas the light chain makes contacts on helix A and extensive cross-linking interactions on an adjacent HA protomer. Antibody CT149 has also been shown to bury ~250 Å^2^ of the same adjacent protomer^32^; however, compared with 3I14, CT149 has vast differences in overall HA recognition. CT149 approaches HA at a different angle, which permits light-chain contacts with HA1, whereas the heavy chain makes fewer contacts within the stem hydrophobic groove (Supplementary Fig. 9).

An engineered LCDR3 point mutant, 3I14^D93N^, shows a sevenfold increase in binding affinity towards H6, while retaining binding to H10 and H3. A single hydrogen bond and better charge complementarity between 3I14 and H6 are the driving force for the improvement. Importantly, this mutation could be generated in somatic B cells in vivo by activation-induced deamination (AID) U:G mismatch repair^38^. Similar improved binding affinity for 3I14^D93N^ to H5 (which is highly conserved with H6 at the 3I14 binding interface) also resulted in tenfold higher potency in a pseudotyped virus neutralization assay^27^. This correlation between binding and neutralization makes 3I14^D93N^ a more attractive option for development as a therapeutic or prophylactic for treatment of severe IAV infection, as passive immunization with anti-HA antibodies has shown promising effects^39^. In addition, the 3I14 HCDR3 might be considered as a next-generation scaffold to produce small molecules with cross-group therapeutic potential, such as the cyclic peptidic fusion inhibitors recently reported^40^.

Recognition of the HA stem hydrophobic groove by 3I14 is similar to group-specific and more broadly reactive antibodies from diverse germlines^18,22,23,30^, as well as that from the three other *V*_*H*_*3-30*-derived antibodies that have structures reported (FI6v3, 39.29, and 3.1^24–26^). *V*_*H*_*3-30* bnAbs do not interact with HA using HCDR2, whereas the use of HCDR2 can infer that a germline is inherently fit for pathogen interaction, such as the “IF” portion of the IFY motif in *V*_*H*_*1-69*-derived bnAbs^21,41^. Comparison of 3I14 with FI6v3, 39.29, and 3.1 complexes shows that they each have different HCDR3s and CDR-binding contributions to the hydrophobic groove, different light chains that contribute differently to binding, varying angles of approach, and distinct but overlapping footprints on HA. Despite different developmental pathways, a key similarity for the *V*_*H*_*3-30*-derived bnAbs is a shared V_H_–J_H_ gene segment usage, which can combine with variable D_H_ segments to produce HCDR3 peptides, which preferentially recognize pockets near Trp21. Notably, FI6v3 and 39.29 each contain HCDR3s with defined molecular signatures, grouping them with the FGV/I motif (*V*_*H*_*1-18*), and the LXYFXWL motif (*V*_*H*_*6-1*), respectively. The ability for *V*_*H*_*3-30*-derived bnAbs to develop a range of signatures that are shared with divergent germlines provides additional insights towards the plasticity of this V-segment scaffold.

Similar to many stem-directed antibodies, 3I14 provides protection from influenza through Fab- and Fc-mediated interactions^27^. In a mouse model, Fc-mediated effector functions are required for protection from influenza by both stem- and head-directed antibodies^42^. However, not all antibodies providing a protective effect are found to neutralize the virus in vitro^18,42^, suggesting that screening based on heterotypic binding and in vitro neutralization may fail to identify antibodies providing the greatest protective effect. Shifting the focus from bnAbs to broadly protective antibodies will be important when evaluating the effectiveness of a vaccination strategy or to identify antibodies with therapeutic potential.

Antibodies targeting conserved epitopes on the HA stem are an important part of the immune response to influenza infection and vaccination^43,44^. However, in either case, the immune system has a preference for non-stalk epitopes, thus posing an immunological obstacle towards universal vaccine efforts^45,46^. This might be overcome through thoughtful antigen design and heterologous prime boost vaccination strategies, such as the headless and chimeric HAs, which are showing promise in eliciting stem-specific immune responses in preclinical settings^47–53^. Another possibility might be germline-targeted immunogens, such as those being developed for HIV^54–56^, to activate germline precursors, which could then be expanded upon with subsequent vaccinations. The structural definition of the 3I14 epitope adds to the growing knowledge base of antigenic determinants necessary for eliciting antibodies, which have the ability to confer protection against circulating and emerging strains of IAV and should provide new insights to ongoing universal vaccine design efforts.

## Methods

### Fab expression and purification

3I14 and 3I14^D93N^ Fabs were individually cloned into pFastBac Dual vectors (Invitrogen) with an N-terminal honeybee melittin signal peptide fused to the light chain and a GP64 signal peptide fused to the heavy chain, along with a C-terminal His_6_-tag also fused to the heavy chain. Recombinant bacmid DNA was generated using the Bac-to-Bac system (Invitrogen) and baculovirus was generated by transfecting purified bacmid DNA into Sf9 cells using Cellfectin II (Invitrogen). Fabs were expressed by infecting suspension cultures of High Five cells (Invitrogen) with baculovirus shaking at 110 r.p.m. for 72 h at 28 °C. The proteins were purified by Ni-NTA (Qiagen), followed by gel filtration (GE Healthcare) in 10 mM HEPES pH 7.5, 150 mM NaCl, and 5% glycerol.

### Expression and purification of HAs for crystallization

The HA0’s from H3 (A/Victoria/361/2011), H6 (A/Taiwan/2/13), and H10 (A/Jiangxi-Donghu/346/2013) were codon optimized and cloned into pFastBac vectors for expression in High Five insect cells. All constructs were fused with an N-terminal gp64 signal peptide, a C-terminal thrombin cleavage site, followed by a foldon trimerization sequence, and a His_6_-tag, as also described previously^57^. HA0s were purified via His-tag affinity chromatography, digested overnight with trypsin (New England Biolabs, 5 mU trypsin per mg HA, 16 h at room temperature) to remove the His-tag/trimerization domain and ensure a uniform population of HA1/HA2. HAs were then run over a gel filtration column and found to be >95% pure by SDS-polycrylamide gel electrophoresis (PAGE) analysis.

### Crystallization and structural determination of HA-Fab complexes

For 3I14 Fab-HA or 3I14^D93N^Fab-HA complex formation, Fab was added to HA in a molar ratio of 3.5 : 1, to achieve three Fabs per HA trimer, incubated at room temperature for 1 h or overnight at 4 °C, and the complex was then purified from unbound Fab by gel filtration in buffer containing 10 mM HEPES pH 7.5, 150 mM NaCl, and 5% glycerol. Protein complexes were concentrated to 10 mg/mL and sitting drop vapor diffusion screens for all complexes were set up using a Phoenix liquid handler (Art Robbins Instruments) at a protein to buffer ratio of 1 : 1 and stored at 20 °C.

H3-3I14 Fab crystals appeared within 3–5 days and were optimized via the hanging drop method in buffer containing 18–24% w/v PEG 3350, 0.2 M KCN, and 0.1 M Bis-Tris-Propane pH 7.5. Crystals were cryoprotected in mother liquor supplemented with 20% glycerol, flash-cooled in liquid nitrogen. X-ray diffraction data were collected to 3.7 Å resolution at the 19BM-D beamline (wavelength 0.97919 Å) at the Advanced Photon Source (APS) and were processed in space group *P*6_3_ using XDS^58^. The structure was determined by molecular replacement with Phaser^59^, using the following search models: (i) a homology model of the variable region of 3I14 built using the PIGs server^60^, which had the HCDR3 removed; (ii) the Fab constant region from PDB 4PY8; and (iii) a single H3 protomer from PDB 4O58. One HA protomer (HA1/HA2) of the HA trimer was found in the asymmetric unit, along with one 3I14 Fab. After initial rounds of rigid body refinement with Phenix^61^, unbiased density from the *F*_o_–*F*_c_ map was used to build the 3I14 Fab HCDR3, as well as for building of N-linked glycans to H3. The model was iteratively rebuilt for several more rounds using Coot^62^ and refined using Phenix, with final *R*_work_/*R*_free_ values of 0.231/0.282, respectively.

H3-3I14^D93N^ Fab complex was found to crystalize in the same condition as native 3I14 Fab-H3 described above. Crystals were cryoprotected in mother liquor supplemented with 20% glycerol, flash-cooled in liquid nitrogen. X-ray diffraction data were collected to 3.3 Å resolution at the 19BM-D beamline (wavelength 0.97919 Å) at APS and were processed in space group *P*6_3_ using XDS^58^. The structure was determined by molecular replacement with Phaser using the solved H3-3I14 structure as a search model, with the mutant D93N residue of 3I14 omitted. The model was refined with Phenix, and residue N93 and glycans were built using Coot. Iterative rounds of refinement resulted in *R*_work_/*R*_free_ values of 0.232/0.281, respectively.

H6-3I14^D93N^ Fab complex crystals appeared in 3–5 days from a drop containing 0.1 M MIB Buffer (sodium malonate, imidazole, and boric acid in the molar ratios 2 : 3 : 3) pH 5.0 and 25% PEG 1500. Crystals were cryoprotected in mother liquor supplemented with 20% glycerol and flash-cooled in liquid nitrogen. X-ray diffraction data were collected to 3.8 Å resolution at the 19BM-D beamline (wavelength 0.97919 Å) at APS and were processed in space group *R*32 using XDS. The structure was determined by molecular replacement with Phaser, using a single protomer of H6 from PDB 4XKD, and the solved portion of 3I14^D93N^Fab from the complex with H3. One HA protomer (HA1/HA2) of the HA trimer was found in the asymmetric unit, along with one 3I14^D93N^Fab. Iterative rounds of reciprocal space and real-space refinement were carried out using Phenix and Coot, respectively, resulting in *R*_work_/*R*_free_ values of 0.276/0.333, respectively.

H10-3I14 Fab complex crystals appeared in 3–5 days from a drop containing 15% w/v PEG 4000, 0.15 M ammonium sulfate, 0.1 M MES pH 6. Crystals were cryoprotected in mother liquor supplemented with 20% glycerol and flash-cooled in liquid nitrogen. X-ray diffraction data were collected to 4.2 Å resolution at the 23ID-B beamline (wavelength 1.033202 Å) at APS and were processed in space group *P*321 using HKL3000^63^. The structure was solved by molecular replacement in Phaser, using a single protomer of H10 from PDB 5TGV as one search model and the solved 3I14 Fab structure from the above H3 complex as the other search model. Real-space and reciprocal space refinement were carried out using Phenix and Coot, with final *R*_work_/*R*_free_ values of 0.261/0.309, respectively.

X-ray data collection and refinement statistics are listed in Table 1. Structural illustrations were prepared using PyMol^64^. Antibody–antigen interface analysis was carried out using the Protein Interfaces, Surfaces and Assemblies software, PISA, obtained from the European Bioinformatics Institute^65^. Prism 7.0 was used for graphical representation to compare buried surface area, CDR lengths and SHMs.

### Expression and purification of 3I14 and 3I14^D93N^ IgG antibodies

Gene fragments for either 3I14 or 3I14^D93N^ were separately subcloned into human IgG1 expression vector TCAE6^66^. The IgG1s were expressed in 293F cells (ThermoFisher, catalog number R79007) by transient transfection and purified by protein A sepharose affinity chromatography followed by buffer exchange into phosphate-buffered saline (PBS) using amicon ultra centrifugal filters with a 50 K molecular weight cut-off.

### Biolayer interferometry

Kinetic analyses of 3I14 and 3I14^D93N^ binding to recombinant HAs were performed on biolayer interferometry using an OctetRED96 instrument (ForteBio, Menlo Park, CA) at 25 °C. The 3I14 and 3I14^D93N^ IgG were diluted to 5 nM in Pierce protein-free blocking buffer (PBS with 0.5% (v/v) Tween-20) and then captured onto anti-human IgG Fc (AHC) biosensors (Fortebio) for 180 s. Binding of recombinant full-length HAs were probed at seven concentrations that were serial diluted starting at 100 nM for H6 experiments and 50 nM for H10 experiments. All experiments contained an additional anti-human IgG Fc antibody biosensor that tested for potential nonspecific interactions between HAs and anti-human IgG Fc. For the measurement of the association rate constant (*K*_on_), association of 3I14 was measured for 300 or 600 s, and for the measurement of *K*_off_, dissociation of 3I14 IgG1 was measured for 700 or 1200 s. Data were reference-subtracted and aligned with each other in the Octet Data Analysis software v11.0 (FortéBio) using a 1 : 1 binding model. All binding traces and curves used for fitting are reported in Supplementary Fig. 1.

### Extrinsic fluorescence assay

Experiments were carried out as described for high-throughput thermal scanning^67^. Specifically, SYPRO Orange dye (Invitrogen) was supplied in dimethylsulfoxide at 5000× the working concentration for PAGE staining. Samples of 20 μL per well were prepared by mixing 1 μL of 200× SYPRO Orange (final concentration 10×) with 19 μL of protein (0.1 mg/mL or 2.1 μM) in PBS. Spectra were obtained on a Bio-Rad CFX96 thermal cycler Real-Time Detection System. Thermal denaturation curves (ramp rate of 1 °C/min at 0.2 °C intervals with an equilibration of 5 s at each temperature before measurement) were acquired by measuring fluorescence intensities using the Förster resonance energy transferchannel with excitation from 450 to 490 nm and detection from 560 to 580 nm. Melting temperatures are reported as the average, ±SD from four independent measurements.

### Reporting summary

Further information on research design is available in the Nature Research Reporting Summary linked to this article.

## Supplementary information

Supplementary Information

Peer Review File

Reporting Summary

## Data Availability

The atomic coordinates and corresponding structure factors have been deposited into the Protein Data Bank with PDB accession codes 6WF1, 6WF0, 6WEX, and 6WEZ. Structures used for molecular replacement can be found under PDB accession codes 4PY8, 4O58, 4XKD, and 5TGV. Structures used for comparison and analysis can be found under PDB accession codes 4KVN, 3ZTJ, 4PY8, 4FQI, 5WKO, 5JW4, 5K9K, 5K9O, 6E3H, 5KAN, and 5KAQ. Other data are available from the corresponding authors upon reasonable request. Source data are provided with this paper.

## Source data

Source Data
