# Supplementary material for: Unique structural solution from a VH3-30 antibody targeting the hemagglutinin stem of influenza A viruses
Source: Nat Commun. 2021 Jan 25;12:559. doi: 10.1038/s41467-020-20879-6 (PMC7835374; doi:10.1038/s41467-020-20879-6)
Supplement: Supplementary file 1 — Supplementary Information [file 41467_2020_20879_MOESM1_ESM.pdf]

Supplementary Information

Unique structural solution from a VH3-30 antibody targeting the hemagglutinin stem of influenza A viruses

Wayne D. Harshbarger*[1], Derrick Deming*[1], Gordon J. Lockbaum[2], Nattapol Attatippaholkun[1], Maliwan Kamkaew[1], Shurong Hou[2], Mohan Somasundaran[2], Jennifer P. Wang[3], Robert W. Finberg[3], Quan Karen Zhu[1], Celia A. Schiffer[#2] and Wayne A. Marasco[#1,4]

[1]Department of Cancer Immunology & Virology, Dana-Farber Cancer Institute, Boston, MA, USA

[2]Department of Biochemistry and Molecular Pharmacology, University of Massachusetts Medical School, Worcester, MA, USA

[3]Department of Medicine, University of Massachusetts Medical School, Worcester, MA, USA

[4]Department of Medicine, Harvard Medical School, Boston, MA, USA

* - These authors contributed equally to this work
# - Corresponding authors:

[#] Celia A. Schiffer, Ph.D.
Director, Institute of Drug Resistance
Department of Biochemistry and Molecular Pharmacology
University of Massachusetts Medical School
364 Plantation Street
Worcester MA 01605-2324
Celia.schiffer@umassmed.edu

[#]Wayne A. Marasco, M.D., Ph.D.
Department of Cancer Immunology & Virology
Dana-Farber Cancer Institute
Harvard Medical School
450 Brookline Avenue
Boston, MA 02215
wayne_marasco@dfci.harvard.edu

a

H10N8 (A/Jiangxi-Donghu/346/2013)

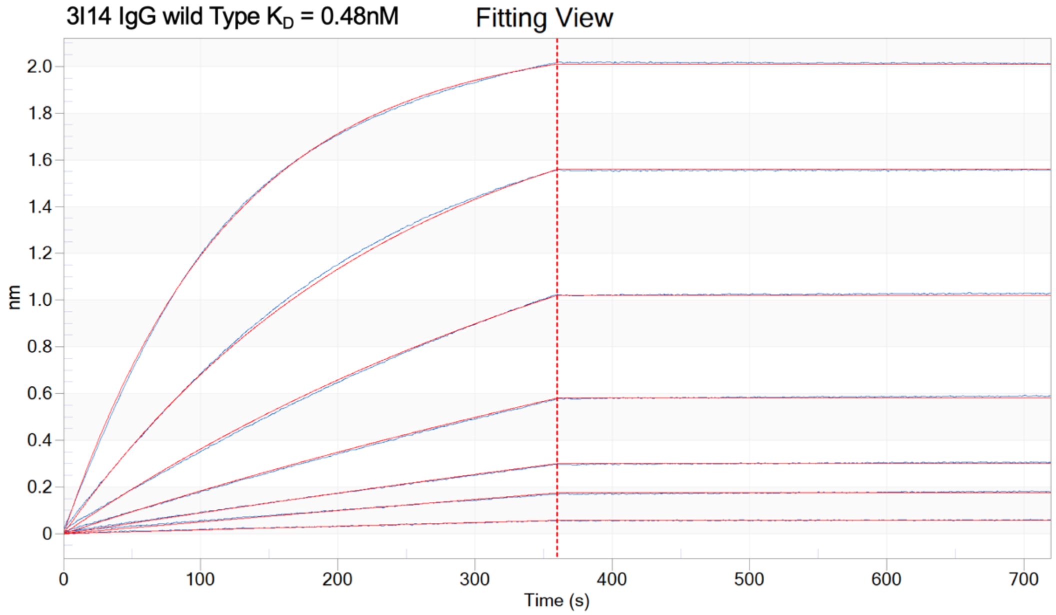

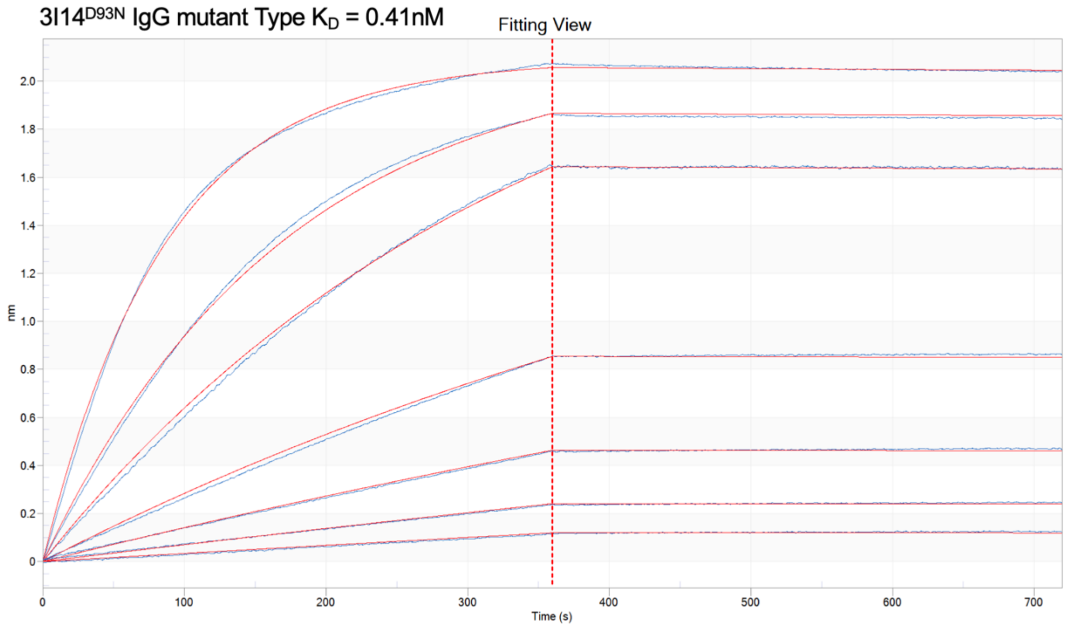

b

H6N1(A/Taiwan/2/2013)

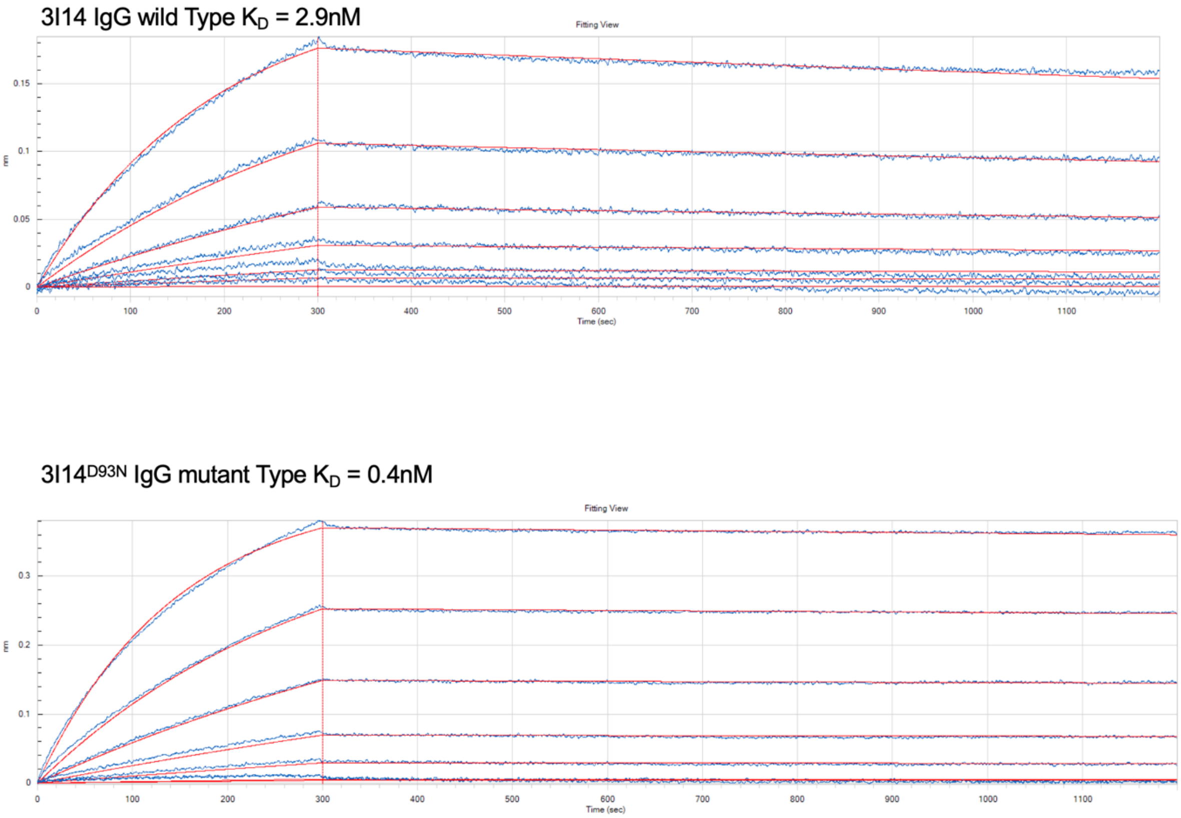

Supplementary Figure 1. 3I14 wild type and VLD93N IgG1 variants binding to recombinant H6 **(a)** and H10 **(b)**. Green or blue curves are the experimental trace obtained from biolayer interferometry experiments, and red curves are the best global fits to the data used to calculate the K_D values. Affinity measurements (K_D values) for the binding curves were reported in Table 1. Source data file available for this figure.

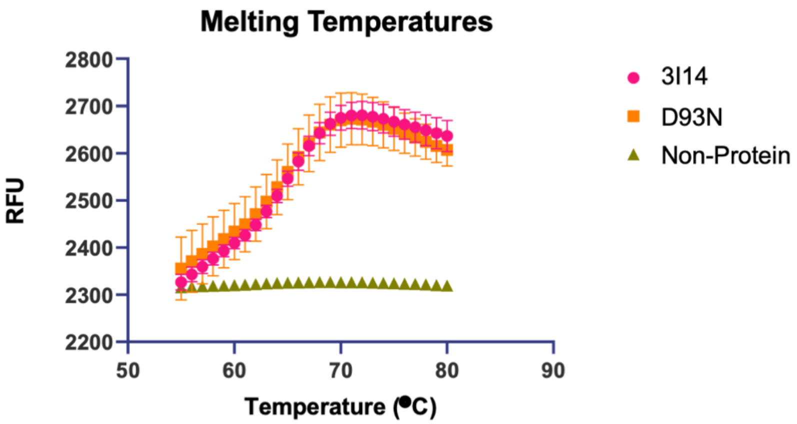

Supplementary Figure 2. Extrinsic fluorescence melting curves for 3I14 IgG, 3I14^{D93N} IgG, and a control non-protein. The melting temperature is constant between 3I14 IgG and 3I14^{D93N} IgG signifying that the mutation does not impact the thermal stability. Error bars mean ± standard deviation from four independent experiments. Source data file available for this figure.

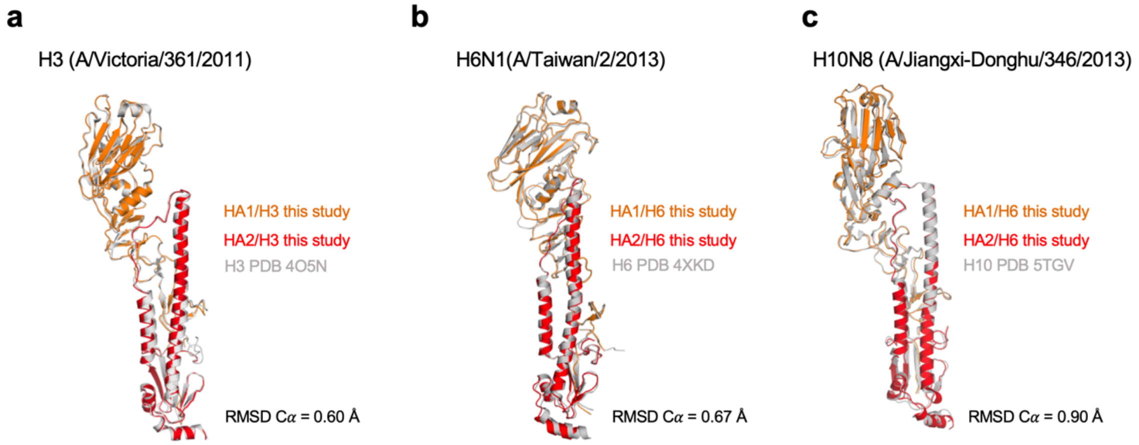

Supplementary Figure 3. Structural comparison of HAs from this study with previously deposited structures. **(a)** H3 compared to PDB 405N; **(b)** H6 compared to PDB 4XKD; **(c)** H10 compared to PDB 5TGV. Root mean square deviation (RMSD) of the C_α atoms for entire HA (HA1/HA2) are shown.

a

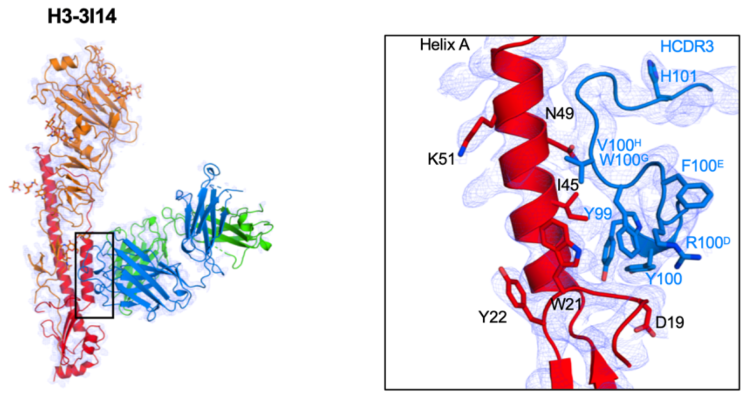
H3-3I14
Helix A
HCDR3
H101
N49
K51
V100H
W100G
F100E
I45
Y99
R100D
Y100
Y22
W21
D19

b

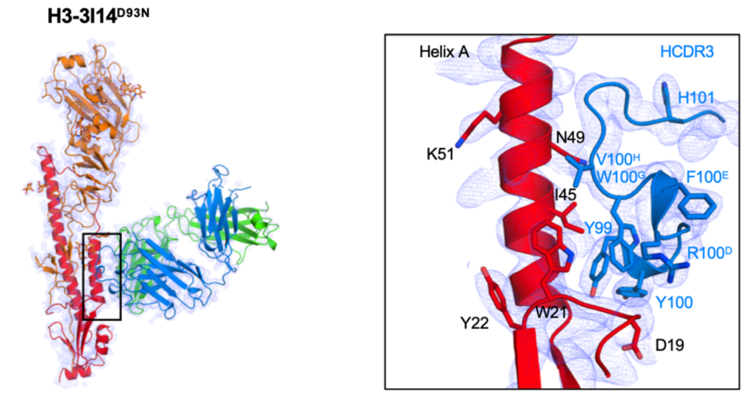
H3-3I14D93N
Helix A
HCDR3
H101
K51
N49
V100H
W100G
F100E
I45
Y99
R100D
Y100
Y22
W21
D19

c

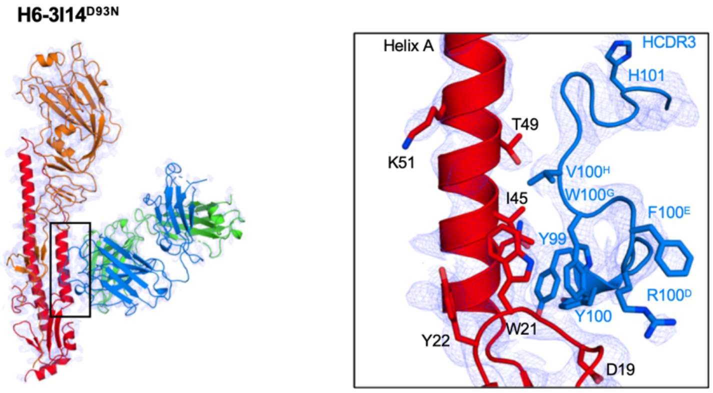

d

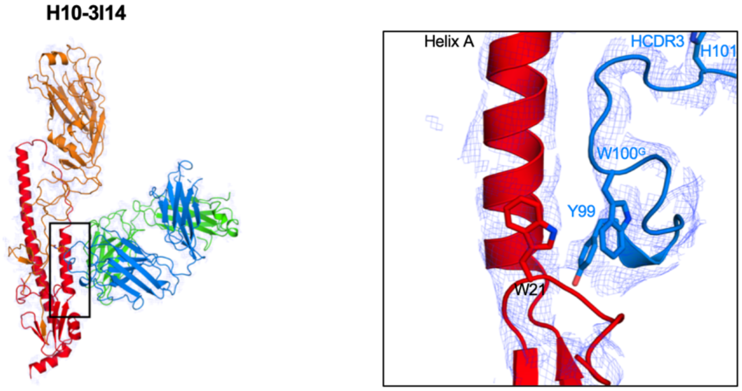

Supplementary Figure 4. Electron density maps scaled at 1 σ for asymmetric units with zoomed view of the 3I14 HCDR3 interaction with helix A for **(a)** H3-3I14; **(b)** H3-3I14^{D93N}; **(c)** H6-3I14^{D93N}; and **(d)** H10-3I14.

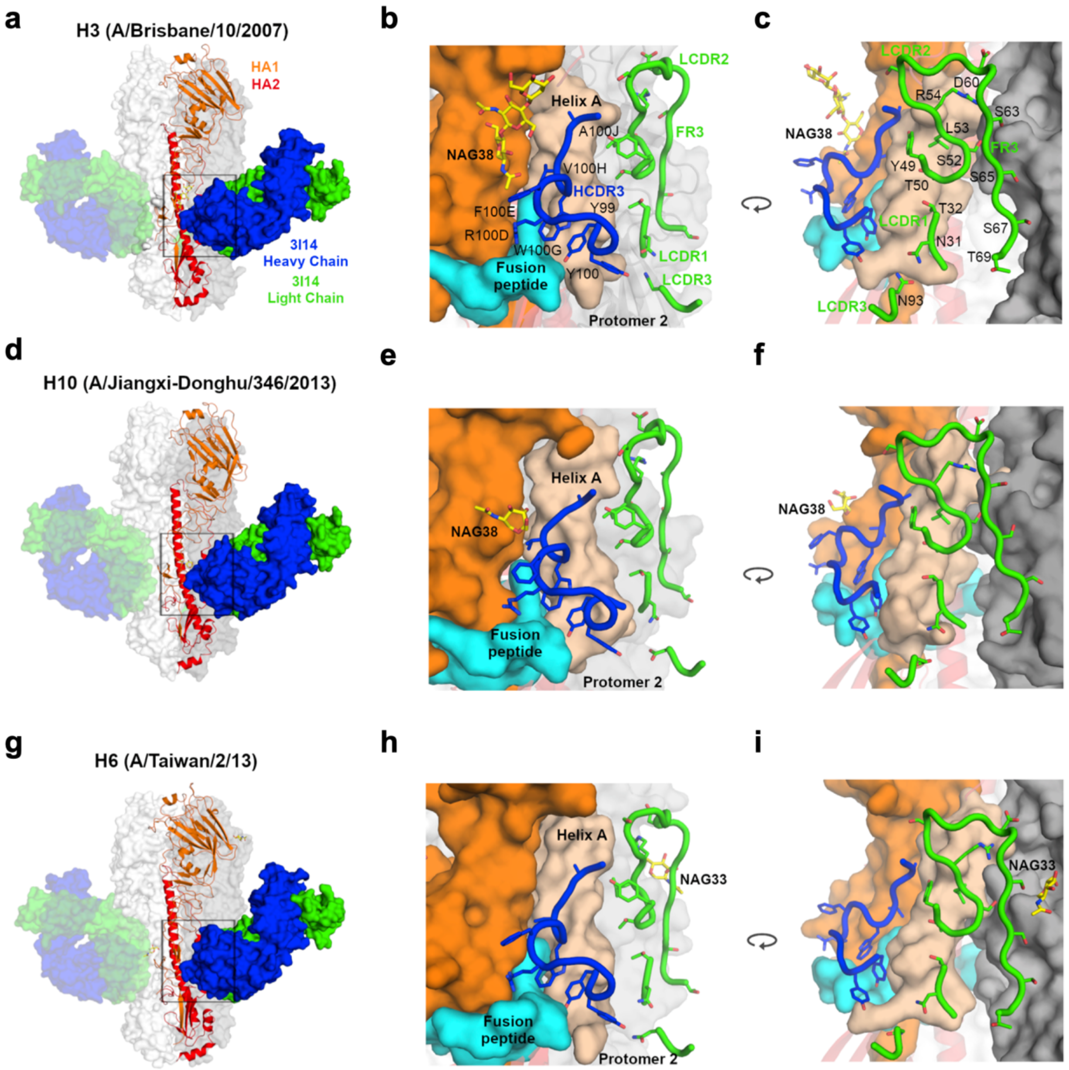

Supplementary Figure 5. Trimeric structures of **(a)** H3-3I14, **(b)** H10-3I14, and **(c)** H6-3I14^{D93N}. HA1 is colored orange, HA2 is colored red, the 3I14 heavy chain is colored blue, and 3I14 light chain is colored green. Panels (b-c, e-f, and h-i) show two different views of the 3I14 heavy and light chain interactions for each complex. Residues for 3I14 are labeled in (b-c).

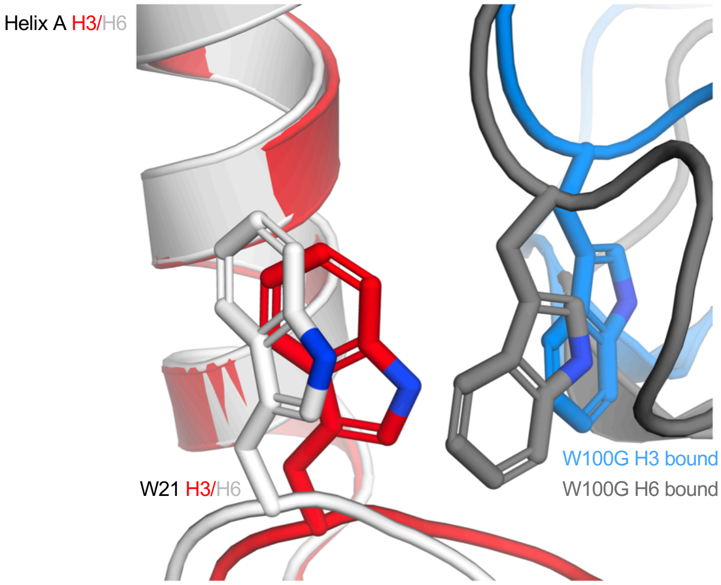

Supplementary Figure 6. Comparison of the proximity of Trp21 for either H3 or H6 to the 3I14 HCDR3 residue Trp100G. HA from H3 is colored red with the bound 3I14 colored blue, while HA from H6 is colored white with bound 3I14 colored dark grey. Structures were aligned by C_α atoms of the HA2 domains, resulting in an RMSD of 1.6 Å.

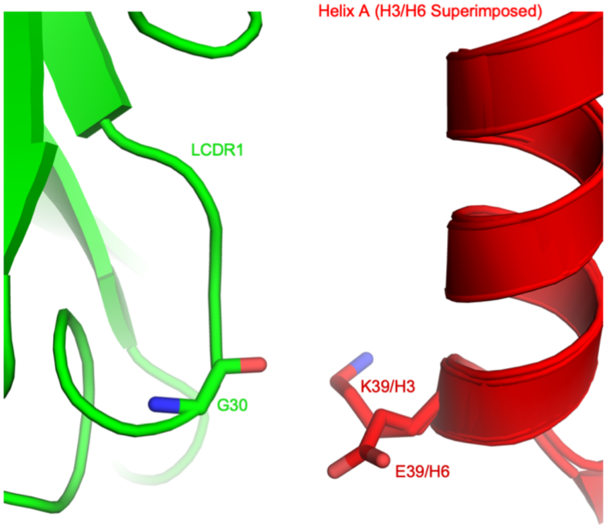

Supplementary Figure 7. Contact interface for 3I14 LCDR1 somatic hypermutation Gly30 with HA from group 1 or group 2 (superimposed). The Gly30 SHM from a germline serine prevents the serine polar side chain from interacting with the hydrophobic side chains of either Ly39 (group 2) or Glu39 (group 1).

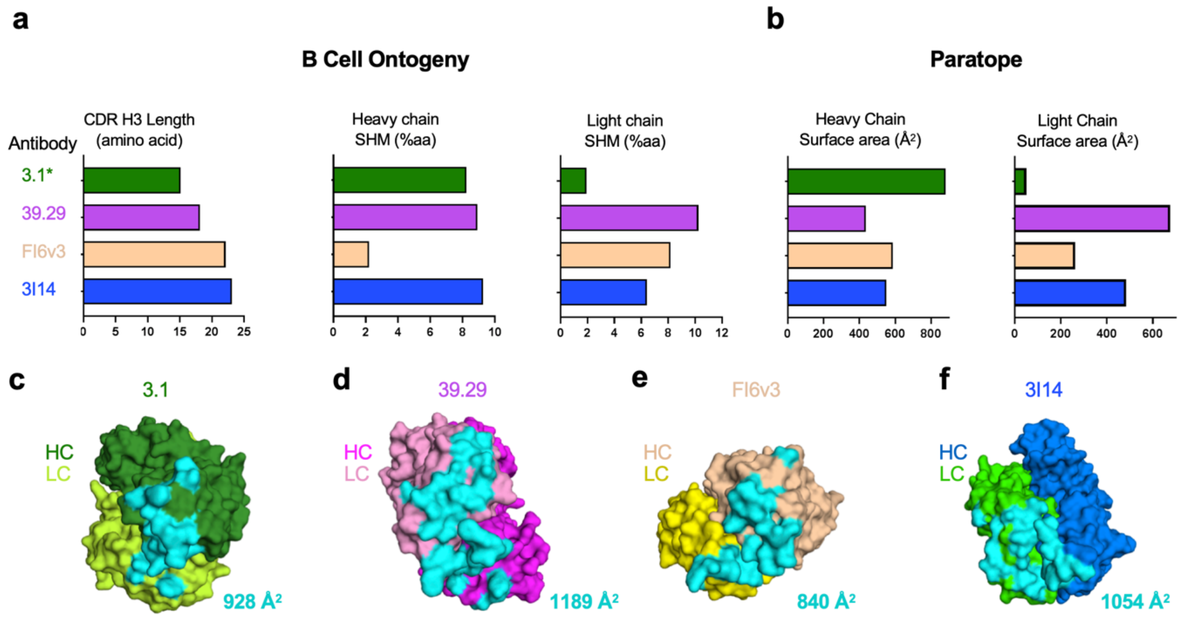

Supplementary Figure 8. B cell ontogeny and paratope characteristics of *V_H3-30* bnAbs. (a) Bar graph representation of an interrogation of the B cell ontogeny for each *V_H3-30* bnAb based on HCDR3 length, and heavy and light chain SHMs. The asterisk indicates that mAb 3.1 was selected from phage display. **(b)** Bar graphs depicting the BSA on HA for heavy and light chains from each antibody. **(c-f)** Paratope view for each antibody. The antibodies are shown in surface representation and heavy and light chains are colored as labeled in the figure. Paratope buried surface areas are colored cyan.

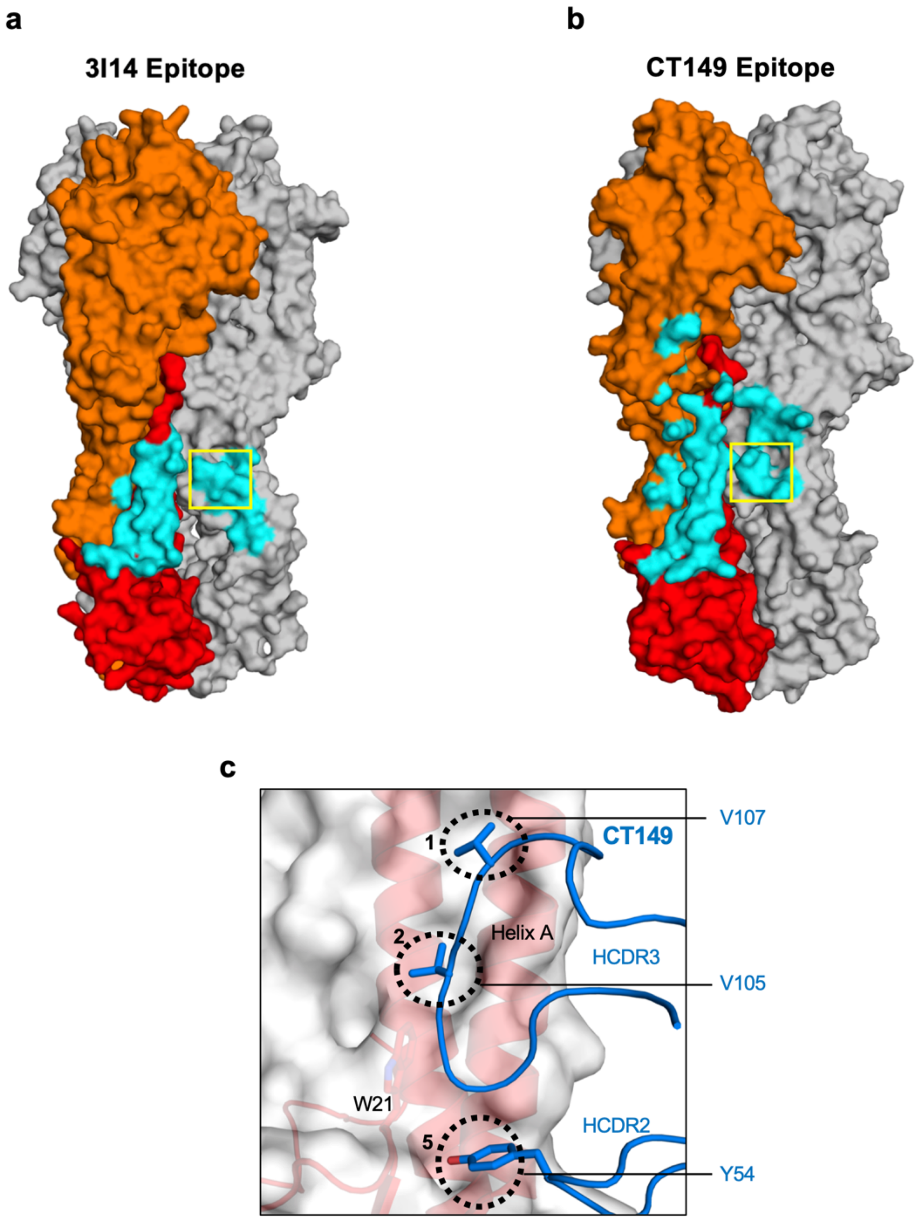

Supplementary Figure 9. Epitope comparison between 3I14 and CT149. The HA trimer is shown in surface representation with the primary protomer targeted by the 3I14 **(A)** or CT149 (PDB 4R8W) **(B)** colored as orange for HA1 and red for HA2, and the adjacent protomers are colored as grey. The epitopes targeted by 3I14 or CT149 are colored in cyan. A yellow box indicates the portion of cross-protomer contacts that are shared between the two antibodies. **(C)** Antibody CT149 interactions with the HA hydrophobic groove. HA2 is shown as a red cartoon with white surface. CT148 heavy chain CDRs which make contacts in the groove are shown as blue cartoon with residues that contact the groove shown as sticks.
