# Supplementary material for: Unique structural solution from a VH3-30 antibody targeting the hemagglutinin stem of influenza A viruses
Source: Nat Commun. 2021 Jan 25;12:559. doi: 10.1038/s41467-020-20879-6 (PMC7835374; doi:10.1038/s41467-020-20879-6)
Supplement: Supplementary file 3 — Reporting Summary [file 41467_2020_20879_MOESM3_ESM.pdf]

nature research

Corresponding author(s): Wayne A Marasco, Celia Schiffer

Last updated by author(s): Nov 21, 2020

Reporting Summary

Nature Research wishes to improve the reproducibility of the work that we publish. This form provides structure for consistency and transparency in reporting. For further information on Nature Research policies, see our Editorial Policies and the Editorial Policy Checklist.

Statistics

For all statistical analyses, confirm that the following items are present in the figure legend, table legend, main text, or Methods section.

n/a	Confirmed	
☐	☒	The exact sample size (*n*) for each experimental group/condition, given as a discrete number and unit of measurement
☐	☒	A statement on whether measurements were taken from distinct samples or whether the same sample was measured repeatedly
☒	☐	The statistical test(s) used AND whether they are one- or two-sided<br>*Only common tests should be described solely by name; describe more complex techniques in the Methods section.*
☒	☐	A description of all covariates tested
☒	☐	A description of any assumptions or corrections, such as tests of normality and adjustment for multiple comparisons
☐	☒	A full description of the statistical parameters including central tendency (e.g. means) or other basic estimates (e.g. regression coefficient) AND variation (e.g. standard deviation) or associated estimates of uncertainty (e.g. confidence intervals)
☒	☐	For null hypothesis testing, the test statistic (e.g. *F*, *t*, *r*) with confidence intervals, effect sizes, degrees of freedom and *P* value noted<br>*Give P values as exact values whenever suitable.*
☒	☐	For Bayesian analysis, information on the choice of priors and Markov chain Monte Carlo settings
☒	☐	For hierarchical and complex designs, identification of the appropriate level for tests and full reporting of outcomes
☒	☐	Estimates of effect sizes (e.g. Cohen's *d*, Pearson's *r*), indicating how they were calculated

Our web collection on statistics for biologists contains articles on many of the points above.

Software and code

Policy information about availability of computer code

Data collection	HKL3000, OctetRed96 (ForteBio), Bio-Rad CFX96 thermal cycler Real-Time Detection System (Bio-Rad)
Data analysis	HKL3000, XDS (version Jan 31, 2020), ForteBio Data Analysis 11.0, Phenix 1.17.1, Coot 0.8.9.2, Prism 7.0, Octet Data Analysis v. 9.0

For manuscripts utilizing custom algorithms or software that are central to the research but not yet described in published literature, software must be made available to editors and reviewers. We strongly encourage code deposition in a community repository (e.g. GitHub). See the Nature Research guidelines for submitting code & software for further information.

Data

Policy information about availability of data

All manuscripts must include a data availability statement. This statement should provide the following information, where applicable:

- Accession codes, unique identifiers, or web links for publicly available datasets
- A list of figures that have associated raw data
- A description of any restrictions on data availability

Atomic coordinates and structure factors for the H3-3I14 Fab, H3-3I14D93N Fab, H6-3I14D93N Fab, and H10-3I14 Fab complexes have been deposited with the Protein Data Bank under accession codes 6WF0, 6WEZ, 6WEX, and 6WF1, respectively. Structures used for molecular replacement can be found under PDB accession codes 4PY8, 4O58, 4XKD, 5TGV. Structures used for comparison and analysis can be found under PDB accession codes 4KVN, 3ZTJ, 4PY8, 4FQI, 5WKO, 5JW4, 5K9K, 5K9O, 6E3H, 5KAN, and 5KAQ. Source data are provided with this paper for Supplementary Figures 1 and 2. Other data are available from the corresponding authors upon reasonable requests.

Field-specific reporting

Please select the one below that is the best fit for your research. If you are not sure, read the appropriate sections before making your selection.

[x] Life sciences [ ] Behavioural & social sciences [ ] Ecological, evolutionary & environmental sciences

For a reference copy of the document with all sections, see nature.com/documents/nr-reporting-summary-flat.pdf

Life sciences study design

All studies must disclose on these points even when the disclosure is negative.

Sample size	We did not perform sample size calculation as our aim was to obtain structural information on the known antibody, 3I14, in complex with its epitope. We also performed additional experiments to understand the binding affinity to hemagglutinin from different strains of influenza A.
Data exclusions	No data were excluded from this analysis.
Replication	Extrinsic fluorescence experiments are the average of four independent experiments. BLI binding experiments/values are derived from a single experiment. All attempts at replication resulted in similar results. Structural data was performed once and proper validation confirmed the validity of the structure.
Randomization	No randomization was applicable to the study as we wanted to assess the structural and biological mechanism of a known antibody.
Blinding	No blinding was applicable to the study as we specifically studied known antibodies.

Reporting for specific materials, systems and methods

We require information from authors about some types of materials, experimental systems and methods used in many studies. Here, indicate whether each material, system or method listed is relevant to your study. If you are not sure if a list item applies to your research, read the appropriate section before selecting a response.

Materials & experimental systems

n/a	Involved in the study	
[ ]	[x]	Antibodies
[ ]	[x]	Eukaryotic cell lines
[x]	[ ]	Palaeontology and archaeology
[x]	[ ]	Animals and other organisms
[x]	[ ]	Human research participants
[x]	[ ]	Clinical data
[x]	[ ]	Dual use research of concern

Methods

n/a	Involved in the study	
[x]	[ ]	ChIP-seq
[x]	[ ]	Flow cytometry
[x]	[ ]	MRI-based neuroimaging

Antibodies

Antibodies used	3I14 and 3I14-D93N IgG1 and Fab were produced in house. The 3I14 and 3I14D93N IgG1 were diluted to 5 nM in Pierce protein-free blocking buffer (PBS with 0.5% (v/v) Tween-20) for binding studies. For BLI studies, anti-human IgG Fc (AHC) biosensors were used (Fortebio, catalog number 18-5060).
Validation	Validation for these antibodies was done by binding studies as well as by the structural information obtained.

Eukaryotic cell lines

Policy information about cell lines

Cell line source(s)	FreeStyle-293F cells (Invitrogen), SF9 (ThermoFisher), High Five (ThermoFisher)
Authentication	No cell lines were authenticated. All cells were purchased commercially and are not misidentified.
Mycoplasma contamination	All cells have tested negative for mycoplasma contamination.
Commonly misidentified lines (See ICLAC register)	No commonly misidentified cell lines have been used in this study.
