# Supplementary material for: Unique structural solution from a VH3-30 antibody targeting the hemagglutinin stem of influenza A viruses
Source: Nat Commun. 2021 Jan 25;12:559. doi: 10.1038/s41467-020-20879-6 (PMC7835374; doi:10.1038/s41467-020-20879-6)
Supplement: Supplementary file 2 — Peer Review File [file 41467_2020_20879_MOESM2_ESM.pdf]

REVIEWER COMMENTS

Reviewer #1 (Remarks to the Author):

Marasco et al have recently reported discovery and in vivo characterization of VH3-30 derived human antibody 3I14 that neutralize group 1 & group 2 influenza A viruses (Fu Y. et al. Nat Commun 7, 12780, 2016). In the present study, the authors have extended their study by elucidating structural basis for neutralization of influenza virus by solving crystal structures of 3I14 Fab in complex with influenza hemagglutinin (HA) from group 1 (H6), and group 2 (H3 and H10) HAs. In comparison with known VH3-30 derived broadly neutralizing antibodies (bnAbs) such as FI6v3, 39.29 and 3.1, 3I14 shows a similar binding mode in the HA stem region with slightly different angle of approach and molecular interactions. Like FI6v3, 3I14 predominantly uses a heavy chain complementary determining region loop (HCDR3) to interact with the hydrophobic cavity in stem region of HA and completely occupies this region. Thus, structural data presented here may provide a good starting point for the design and development of broad peptide or small molecule based therapeutic against group 1 and group 2 influenza A viruses. The data are well presented and represent a very interesting comparison of VH3-30 antibodies directed to the HA stem. It is still not clear why VH3-30 is selected and the discussion in the paper about VDJ recombination warrants more discussion as to why VH3-30 as distinct from other germlines is used here.

Other comments:

Page 4, line 6. “tend to be more robust than head-directed antibodies” Can we please indicate what ' robust' means as it does not really relate to breadth.

Page 4, lines 4-5 from bottom of page. Please replace “stem directed” with “stem-directed”.

Page 5, line 9. Please replace “provide” with “provides”.

Page 5, line 4-5 from bottom of page. Please replace “which is the residue in H5 which led to the engineering of 3I14D93N” with ‘which in H5 led to the engineering of 3I14D93N.

Page 6, line 11. Please replace “fab” with ”Fab”.

Page 6, line 3 from bottom of page. Please replace “complementary” with “complementarity”.

Page 7, line 14. “Interestingly, 3I14 alters its angle of approach” This is in part define below but is there also any change is the angle between the VH and VL in the different complexes?

Page 8, line 16. “Remarkably” I do not know what is remarkable here as other VH3-30 antibodies primarily use CDR H3. There are too many remarkable(s) throughout.

Page 9, lines 1-3 from bottom of page. “The criticality of the HCDR3 shape is thus exemplified by the SHMs which surround it, suggesting that the maturation of 3I14 was driven by a preference for a prepositioning of the HCDR3.” Is the unliganded structure known so one could determine any induced fit?

Page 10, line 13. Please replace “Ly39” with “Lys39”.

Page 10, line 17. “Lys39HA is flipped”. Is it really flipped or just a different rotamer around the amino group?

Page 10, lines 18-19. "Lys39HA allows it to extend into the center of the negative patch formed between Asp93LCDR". What is the distance between the Lys amine and the carboxyl of Asp 93?

Page 11, lines 17-18. "the VH domains for 39.29, FI6v3, and 3.1 are rotated by ~42o, 49o, and 62o, respectively" There also seems to be a difference in tilt as well as rotation and this should be cited?

Page 11, line 3 from bottom of page. Please replace "HA2," with "HA2;".

Page 11, last line on page. Please replace "extend" with "extent".

Page 12, line 4 from bottom of page. Please replace "germline encoded" with "germline-encoded".

Page 13, line 20. "remarkable" Another unwarranted use of 'remarkable'.

Page 13, lines 21-22. "HA but not shed monomeric HA". Is there a reference that cites shedding of monomeric HA as a problem in antibody elicitation?

Page 13, lines 1-2 from bottom of page. "suggesting that plasticity in binding angles of VH3-30-derived antibodies is an important factor for their wide neutralization breadth" As also seen for VH1-69 Abs to the HA stem, please cite.

Page 14, lines 14-15. "a key determinant in the biased selection and expansion of VH3-30 germline antibodies" Are you suggesting that other germline antibodies cannot recombine with these D and J regions or that VH3-30 preferentially does so?

Page 16, lines 7 and 18. Please replace "flash frozen" with "flash cooled".

Page 17, lines 1 and 10. Please replace "flash frozen" with "flash cooled".

Page 20, Reference 32. Incomplete reference.

Page 27, Table 1

Please add the number of unique reflections measured.

Please also add Rpim.

I/σI Please limit to one decimal point.

Please add B values for HA and for antibody.

Reviewer #2 (Remarks to the Author):

Harshbarger et al describe and analyze the structure of the broadly neutralizing influenza HA stem binding antibody 3I14 in complex with H6, H3 and H10. This works builds on earlier work published in 2016 when this antibody was first described and characterized but used in silico modeling to predict antibody structure and docking to H3 HA. This manuscript extends this work by solving the x-ray crystal structure of the 3I14 Fab in complex with both a group 1 and group 2 HAs and comparing the

structure to other VH3-30 HA stem binding antibodies. There are few reports of the same antibody in complex with both a group 1 and group 2 HA and the structural analysis is very detailed and well described. This work gives new insight into how antibodies can accommodate the unique structural elements of both a group 1 and group 2 HA stem. However, some of the conclusions, including claims of a unique VH3-30 molecular signature are not well-founded and should be reconsidered or better substantiated. Comparing the structure of 3I14 not only to other VH3-30 HA stem binding antibodies, but other cross-group binding antibodies would be informative.

Specific Points:

1. This work provides little evidence that VH3-30 derived cross-group HA binding antibodies have enough similarities to be grouped together with an identifiable molecular signature. As described in this manuscript, the four VH3-30 cross-group HA binding antibodies compared here have very different CDRH3s with few residues in common, different LCs with different contributions to binding, different angles of approach, and varying footprints on the HA stem. The claim that targeting two particular pockets surrounding Trp21 points to a unique signature is not convincing as this region is commonly targeting by cross-group HA binding antibodies, not just VH3-30 encoded antibodies. As such it would be informative to compare the structure of 3I14 not only to other VH3-30 encoded antibodies but also to other cross-group HA binding antibodies including those that don't fall into established classes, such as the VH3-23 encoded antibody 31.a.83 (Joyce et al, Cell 2016) which appears to have some similarities to 3I14.

2. While 3I14 is unique among the other three VH3-30 encoded antibodies in the amount of LC cross-protomer interaction, other cross-group antibodies, including CT149 (Wu et al Nat Comm 2015) also have some LC cross-protomer interactions. This should be acknowledged and compared.

Minor point:
On page 7 in the 2nd large paragraph, reference to Supplementary Figure 6 instead of 5 seems most appropriate.

Reviewer #3 (Remarks to the Author):

Harshbarger & Deming together with colleagues report the structural basis for influenza hemagglutinin recognition by human antibody 3I14. This antibody, though using IGHV3*30, engages stem epitopes via a distinctive paratope. They provide a structural rational for this antibody's ability to bind broadly to HAs and why an engineered mutation has enhanced affinity for some group 1 HAs. The work is technically sound and structural statistics are consistent with expert refinement experience.

The manuscript and its findings may be a bit overstated. The results themselves are not particularly novel. 3I14, like other stem-directed antibodies, uses IGHV3*30. Though aspects of its contacts are unique it relies upon a HCDR3 of "unusual shape". I am unsure how this antibody, specifically, informs improvements to an influenza vaccine. Similar antibodies are unlikely to be common in B cell repertoires because of the requirement for a specific IGHV gene together with rearrangements that yield an "unusual" HCDR3 geometry. Selectively eliciting these may prove challenging. Whether the mutation D94N, which enhances affinity to some HAs could reliably occur in vivo is also unclear. Structures of the same Fab with multiple HAs produces the expected result, namely the antibody contacts are similar. Many of the more interesting points come from comparisons with reported structures.

I will emphasize that I find the work expertly performed. It is the context in which they are presented and the current state of the field that tempers this reviewers enthusiasm. Perhaps restating some of the major highlights without an attempt to "sell" the reader on the possibility of another pandemic may be more effective.

Major:
The biological consequences of the flexible angle of approach are unclear and perhaps overstated. As depicted in Figure 1 panels D and E contacts with H3 and H6 are effectively indistinguishable. As presented it is difficult to establish whether the flexible CDRs that contact HA permit certain degrees of local flexibility or whether the observed conformational fluctuations have biological purpose. Data at current are insufficient to distinguish between these possibilities.

The alternative approaches are calculated from the superposition of H3 and H6 HAs. This superposition, panel C, appears to produce local deviations within the 3I14 epitope that propagate through the body of the Fab. Measuring the deviation of the Fabs at their most distal atoms results in an apparent exaggeration of minor conformational fluctuations. Superposition, based upon the epitope and local features may produce more accurate measurements.

It is difficult to discern how much more deeply HCDR3 extends into the hydrophobic pocket of H6 HAs. Measurements and reference points should be stated in the text. Is the "deeper" pocket a consequence of antibody engagement or an inherent feature of H6 HAs? Is the pocket also influenced by how the superposition was performed?

The fusion peptide contacts 3I14. In the structures HA0 has been processed into HA1-HA2. Upon cleavage, the fusion peptide undergoes a conformational rearrangement. Is binding of the Fab reliant upon the rearranged HA1-HA2 form or can it bind the HA0 form as well? Has this been demonstrated?

Is the D94N substitution likely to occur via AID-mediated somatic hypermutation? If the stated advancement is to improve immunogens, reliance on mutations that are unlikely to occur in a B cell would negate the authors' main point.

Interestingly, the remarkable shape of the 3I14 HCDR3 is molded from a series of heavy and light chain somatic hypermutations (SHMs). I do not find this observation to be remarkable. These arrangements are often seen and are implicit in many reported antibodies. Schmidt et al., PNAS, 2013, examined one instance.

The authors should discuss the growing body of evidence indicating that virus neutralization is not the primary mechanism of protection of stem antibodies. (e.g DiLillo et al., 2014). The antibodies in this manuscript were assayed for neutralization over multiple replication cycles and certainly do inhibit replication but they may not be potently neutralizing on a single round of infection. Broadly protective may be a more appropriate term.

Minor:
Line numbers would be quite helpful.

Introduction-There are 18 influenza A HA serotypes

D93N mutation was previously reported as D94N. It is listed both ways in the draft. Which is the correct position?

Language about when viruses emerged or arose in the introduction is confusing. Do the authors use

these terms to indicate when specific lineages were produced by reassortment, acquired specific substitutions or infected humans?

Much of the first section “Binding of 3I14 to human infecting H6 and H10 hemagglutinins” is preempted by the structures that follow. Readability would improve by drastically shortening this section to the “highlights” a reader needs to understand this work.

“Neither of these HAs were included in our original characterization of 3I14, though phylogenetically similar viruses such as H1, H5, H7, and H9 were” The statement is misleading. H10 and H6 are occupy distinct branches on an HA tree. Perhaps consider restating.

“This is only the third time that a bnAb has been solved bound to HAs from both groups”. Because neutralization is not the likely mechanism of protection by these antibodies, listing other examples is appropriate. Broadly protective antibody FluA-20 (Bangaru et al. 2019), though not neutralizing should be added to this list. Neutralizing antibody K03.12 (McCarthy et al., 2018) and a related antibody also had structures solved with H1 and H3 HAs.

Including a panel to a figure showing the stem hydrophobic pockets would be helpful.

Reviewers comments and authors' responses for:

Targeting the Hemagglutinin Stem of Group 1 and 2 Influenza A Viruses: A Novel Solution from a VH3-30 Derived Antibody

By Harshbarger and Deming *et al.*

Reviewer #1

Specific Points

It is still not clear why VH3-30 is selected and the discussion in the paper about VDJ recombination warrants more discussion as to why VH3-30 as distinct from other germlines is used here.

A. We appreciate the reviewers comment and have added to our analysis and discussion around this point.

Line 214: "Altogether, 3I14 represents a novel structural solution among the VH3-30 derived bnAbs, and provides evidence that the VH3-30, D3-22, JH4 combination allows for a unique HCDR3 to recognize an identical location on the HA stem."

Line 238: "In summary, this comprehensive comparison reveals that the V(D)J arrangement for the VH3-30 antibodies can develop multiple signatures to target the HA stem. In some cases, these HCDR3 signatures converge on similar structural solutions that have been utilized by other VH germline segments."

Line 282: "Despite different developmental pathways, a key similarity for the VH3-30 derived bnAbs is a shared VH-JH gene segment usage which can combine with variable DH segments to produce HCDR3 peptides which preferentially recognize pockets near Trp21. Notably, FI6v3 and 39.29 each contain HCDR3s with defined molecular signatures, grouping them with the FGV/I motif (VH1-18), and the LXYFXWL motif (VH6-1), respectively. The ability for VH3-30 derived bnAbs to develop a range of signatures that are shared with divergent germlines provides additional insights towards the plasticity of this V-segment scaffold."

Other comments:

Page 4, line 6. "tend to be more robust than head-directed antibodies" Can we please indicate what ' robust' means as it does not really relate to breadth.

A: We have edited this sentence to read: "Stem-directed broadly neutralizing antibodies (bnAbs), which are elicited by natural infection and vaccination, tend to have wider breadth than head-directed antibodies and are often capable of protecting against entire subtypes, groups, or even types."

Page 4, lines 4-5 from bottom of page. Please replace “stem directed” with “stem-directed”.

A: We have made the correction.

Page 5, line 9. Please replace “provide” with “provides”.

A: We have made the replacement.

Page 5, line 4-5 from bottom of page. Please replace “which is the residue in H5 which led to the engineering of 3I14D93N” with ‘which in H5 led to the engineering of 3I14D93N.

A: We have incorporated this change.

Page 6, line 11. Please replace “fab” with ”Fab”.

A: This has been updated.

Page 6, line 3 from bottom of page. Please replace “complementary” with “complementarity”.

A: We have corrected this misspelling.

Page 7, line 14. “Interestingly, 3I14 alters its angle of approach” This is in part define below but is there also any change is the angle between the VH and VL in the different complexes?

A: We have removed this analysis from the manuscript; however, to answer the Reviewers question, there is no change in the angles between VH and VL among the different complexes.

Page 8, line 16. “Remarkably” I do not know what remarkable here as other VH3-30 antibodies is primarily use CDR H3. There are too many remarkable(s) throughout.

A: We appreciate the Reviewers feedback and agree that this is not a remarkable finding. We have edited to remove the use of “remarkable” throughout the manuscript, including this instance.

Page 9, lines 1-3 from bottom of page. “The criticality of the HCDR3 shape is thus exemplified by the SHMs which surround it, suggesting that the maturation of 3I14 was driven by a preference for a prepositioning of the HCDR3.” Is the unliganded structure known so one could determine any induced fit?

A: We were unable to obtain a structure of the unliganded 3I14 Fab. Because we cannot confirm prepositioning of the HCDR3, we have reworded this section at Line 157 to say, “The large decrease in binding affinity obtained with either heavy or light chain germline reverted

mutants could therefore be due to plasticity of the HCDR3 that may result from the lack of some, or all, of these interactions."

Page 10, line 13. Please replace "Ly39" with "Lys39".

A: We have made the correction.

Page 10, line 17. "Lys39HA is flipped". Is it really flipped or just a different rotamer around the amino group?

A: We have corrected this wording to indicate alternate rotamers.

Page 10, lines 18-19. "Lys39HA allows it to extend into the center of the negative patch formed between Asp93LCDR". What is the distance between the Lys amine and the carboxyl of Asp 93?

A: The distance between the Lys amine and the carboxyl of LCDR3 Asp 93 is 4.1 Angstroms and is 2.4 Angstroms to the carboxy of Asn32. For the 3I14D93N mutant complex with H3, the Lys39 amine group is 2.4 Angstroms from the carboxyl of Asn 93 and 3.4 Angstroms from the carboxyl of Asn 31.

Page 11, lines 17-18. "the VH domains for 39.29, FI6v3, and 3.1 are rotated by ~42°, 49°, and 62°, respectively" There also seems to be a difference in tilt as well as rotation and this should be cited?

A: We have added a comment on the difference in tilt between the antibodies.

Page 11, line 3 from bottom of page. Please replace "HA2," with "HA2;".

A: We have corrected this punctuation.

Page 11, last line on page. Please replace "extend" with "extent".

A: We have fixed this error.

Page 12, line 4 from bottom of page. Please replace "germline encoded" with "germline-encoded".

A: We have updated to include the hyphen.

Page 13, line 20. "remarkable" Another unwarranted use of 'remarkable'.

A: We have removed the word "remarkable".

Page 13, lines 21-22. "HA but not shed monomeric HA". Is there a reference that cites

shedding of monomeric HA as a problem in antibody elicitation?

A: During editing of the discussion we have removed this statement.

Page 13, lines 1-2 from bottom of page. "suggesting that plasticity in binding angles of VH3-30-derived antibodies is an important factor for their wide neutralization breadth" As also seen for VH1-69 Abs to the HA stem, please cite.

A: During editing of the manuscript we have removed this statement.

Page 14, lines 14-15. "a key determinant in the biased selection and expansion of VH3-30 germline antibodies" Are you suggesting that other germline antibodies cannot recombine with these D and J regions or that VH3-30 preferentially does so?

A: We have extended the comparison between V_H3-30 antibodies to include comparison with antibodies that have defined molecular motifs. In doing so, we have removed this statement. However, we do expand on the discussion around the V(D)J usage for V_H3-30 antibodies, such as line 285 "Despite different developmental pathways, a key similarity for the VH3-30 derived bnAbs is a shared VH-JH gene segment usage which can combine with variable DH segments to produce HCDR3 peptides which preferentially recognize pockets near Trp21."

Page 16, lines 7 and 18. Please replace "flash frozen" with "flash cooled".

A: We have made this change.

Page 17, lines 1 and 10. Please replace "flash frozen" with "flash cooled".

A: We have made this change.

Page 20, Reference 32. Incomplete reference.

A: This comment is unclear: however, we have added an additional reference (now references 52-54).

Page 27, Table 1

A: Table one has now become table 2, and we have addressed each of the below comments.

Please add the number of unique reflections measured.

Please also add Rpim.

I/σI Please limit to one decimal point.

Please add B values for HA and for antibody.

Reviewer #2

Specific Points:

1. **This work provides little evidence that VH3-30 derived cross-group HA binding antibodies have enough similarities to be grouped together with an identifiable molecular signature. As described in this manuscript, the four VH3-30 cross-group HA binding antibodies compared here have very different CDRH3s with few residues in common, different LCs with different contributions to binding, different angles of approach, and varying footprints on the HA stem. The claim that targeting two particular pockets surrounding Trp21 points to a unique signature is not convincing as this region is commonly targeting by cross-group HA binding antibodies, not just VH3-30 encoded antibodies. As such it would be informative to compare the structure of 3I14 not only to other VH3-30 encoded antibodies but also to other cross-group HA binding antibodies including those that don't fall into established classes, such as the VH3-23 encoded antibody 31.a.83 (Joyce et al, Cell 2016) which appears to have some similarities to 3I14.**

A: We thank the reviewer for their insights. We have included a new section to compare VH3-30 encoded antibodies with other stem-directed bnAbs which fall into established classes, starting at Line 216.

We have also modified our comparison among the VH3-30 encoded antibodies to conclude that there are not sufficient similarities to group them together. We modified our Results and Discussion to reflect this. We have incorporated this reviewers comments into our Discussion at line 279: "Comparison of 3I14 with FI6v3, 39.29, and 3.1 complexes shows that they each have different HCDR3s and CDR binding contributions to the hydrophobic groove, different light chains that contribute differently to binding, varying angles of approach, and distinct but overlapping footprints on HA."

2. **While 3I14 is unique among the other three VH3-30 encoded antibodies in the amount of LC cross-protomer interaction, other cross-group antibodies, including CT149 (Wu et al Nat Comm 2015) also have some LC cross-protomer interactions. This should be acknowledged and compared.**

A: We thank the reviewer for directing us towards this antibody for comparison. We have acknowledged this in the Discussion and added supplementary figure 10 to compare CT149 binding with that of 3I14.

Minor point:

On page 7 in the 2nd large paragraph, reference to Supplementary Figure 6 instead of 5 seems most appropriate.

A: We have updated this to reflect the correct Supplementary Figure.

<u>Reviewer #3:</u>

<u>Major Comments</u>:

1. The biological consequences of the flexible angle of approach are unclear and perhaps overstated. As depicted in Figure 1 panels D and E contacts with H3 and H6 are effectively indistinguishable. As presented, it is difficult to establish whether the flexible CDRs that contact HA permit certain degrees of local flexibility or whether the observed conformational fluctuations have biological purpose. Data at current are insufficient to distinguish between these possibilities.

The alternative approaches are calculated from the superposition of H3 and H6 HAs. This superposition, panel C, appears to produce local deviations within the 3I14 epitope that propagate through the body of the Fab. Measuring the deviation of the Fabs at their most distal atoms results in an apparent exaggeration of minor conformational fluctuations. Superposition, based upon the epitope and local features may produce more accurate measurements.

A: We thank the reviewer for their analysis and inputs on the binding comparisons. The alignments were carried out by fixing either HA2 for H3 and H6, or fixing only Helix A. In either case, the same result was found (large movement of 3I14), and when performing alignments between the FI6-H3 and FI6-H1 or Medi8852-H5 and Medi8852-H7 structure (with the same alignment strategy), we see less movement between the two FI6 complexes, and even fewer with Medi8852 (measuring from the same residues).

However, we agree that the biological consequences are unclear, and also that the binding of 3I14 to either H3, H6, or H10 is indistinguishable. We also agree that alignments of this nature are very difficult and prone to misinterpretation. Therefore, we have chosen to remove this figure and analysis from the text.

2. **It is difficult to discern how much more deeply HCDR3 extends into the hydrophobic pocket of H6 HAs. Measurements and reference points should be stated in the text. Is the "deeper" pocket a consequence of antibody engagement or an inherent feature of H6 HAs? Is the pocket also influenced by how the superposition was performed?**

A: We have rephrased this sentence to state: "Another contributing factor to effectively neutralizing group 1 and group 2 viruses is the local flexibility of HCDR3 at Trp100G which maintains an ~3-4 Å distance from the group specific orientation of Trp21HA."

We have updated the supplementary figure 6 legend to state that the structures were aligned by C-alpha atoms of the HA2 domains, with an RMSD of 1.6 Å.

3. **The fusion peptide contacts 3I14. In the structures HA0 has been processed into HA1-HA2. Upon cleavage, the fusion peptide undergoes a conformational rearrangement. Is binding of the Fab reliant upon the rearranged HA1-HA2 form or can it bind the HA0**

form as well? Has this been demonstrated?

A: The binding is not reliant on cleavage of HA0. Blocking of cleavage by 3I14 binding to HA0 was demonstrated by Fu et al., 2016, and we have added this to the introduction at Line 47.

4. **Is the D94N substitution likely to occur via AID-mediated somatic hypermutation? If the stated advancement is to improve immunogens, reliance on mutations that are unlikely to occur in a B cell would negate the authors' main point.**

A: We appreciate the reviewers' comment, as this is an important point that should have been addressed. We have updated the Discussion as follows with the appropriate reference:

Line 266: "Importantly, this mutation could be generated in somatic B cells in vivo by activation-induced deamination (AID) U:G mismatch repair."

5. **Interestingly, the remarkable shape of the 3I14 HCDR3 is molded from a series of heavy and light chain somatic hypermutations (SHMs). I do not find this observation to be remarkable. These arrangements are often seen and are implicit in many reported antibodies. Schmidt et al., PNAS, 2013, examined one instance.**

A: We thank the reviewer for their insights and have modified the text to remove "interestingly."

Line 152: "Though HCDR1 and HCDR2 do not directly interact with HA, the SHMs occur at positions that seemingly "mold" the HCDR3 into the conformation needed to recognize the large area within the hydrophobic groove."

6. **The authors should discuss the growing body of evidence indicating that virus neutralization is not the primary mechanism of protection of stem antibodies. (e.g DiLillo et al., 2014). The antibodies in this manuscript were assayed for neutralization over multiple replication cycles and certainly do inhibit replication but they may not be potently neutralizing on a single round of infection. Broadly protective may be a more appropriate term.**

A: We thank the reviewer for this suggestion. We have added to the Discussion at Lines 289-295.

Minor:

Line numbers would be quite helpful.

A: We have now included line numbers.

Introduction-There are 18 influenza A HA serotypes.

A: We have updated this.

D93N mutation was previously reported as D94N. It is listed both ways in the draft. Which is the correct position?

A: We use Kabat numbering in the current work and this results in the residue being at position 93 (opposed to 94 in the original work). We have corrected the typo in the text and clarified in the Introduction.

Language about when viruses emerged or arose in the introduction is confusing. Do the authors use these terms to indicate when specific lineages were produced by reassortment, acquired specific substitutions or infected humans?

A: We have updated the wording in the Introduction at line 48: "A point mutation in the light variable domain, termed 3I14D93N (3I14D94N with prior numbering), was found to increase binding affinity and viral neutralization potential by approximately 10-fold against H5, with no negative effect on binding or neutralization to H3."

Much of the first section "Binding of 3I14 to human infecting H6 and H10 hemagglutinins" is preempted by the structures that follow. Readability would improve by drastically shortening this section to the "highlights" a reader needs to understand this work.

A: We thank the reviewer for this feedback and have edited this section.

"Neither of these HAs were included in our original characterization of 3I14, though phylogenetically similar viruses such as H1, H5, H7, and H9 were" The statement is misleading. H10 and H6 are occupy distinct branches on an HA tree. Perhaps consider restating.

A: We have clarified the wording in this statement at Line 69: "Neither of these HAs were included in our original characterization of 3I14, though phylogenetically similar viruses to H6 (such as H1 and H5) and H10 (such as H7, and H9) were studied."

"This is only the third time that a bnAb has been solved bound to HAs from both groups". Because neutralization is not the likely mechanism of protection by these antibodies, listing other examples is appropriate. Broadly protective antibody FluA-20 (Bangaru et al. 2019), though not neutralizing should be added to this list. Neutralizing antibody K03.12 (McCarthy et al., 2018) and a related antibody also had structures solved with H1 and H3 HAs.

A: We thank the reviewer for this input and have added the suggested references to the text at Line 257.

Including a panel to a figure showing the stem hydrophobic pockets would be helpful.

A: We have added a Figure 7 which compares 3I14 with antibodies from other germlines. This figure is in surface representation and has the pockets indicated.

REVIEWERS' COMMENTS

Reviewer #1 (Remarks to the Author):

The authors have answered almost queries except and have updated the revised manuscript accordingly entitled “Targeting the Hemagglutinin Stem of Group 1 and 2 Influenza A Viruses: A Novel Solution from a VH3-30 Derived Antibody”. The revised manuscript is now suitable for publication.

Minor points to be addressed:

1) Page 12, line 4 from bottom of page. Please replace “germline encoded” with “germline-encoded”.

2) Page 20, Reference 32. Incomplete reference.
A: This comment is unclear: however, we have added an additional reference (now references 52-54).

This reference is incomplete as it does not have a volume (or issue) number.

Please replace

Alberto Cisneros, I., et al. . Role of antibody heavy and light chain interface residues in affinity maturation of binding to HIV envelope glycoprotein. Molecular Systems Design & Engineering, 737-746, doi:doi.org/10.1039/C8ME00080H (2019).

with

Alberto Cisneros, I., et al. Role of antibody heavy and light chain interface residues in affinity maturation of binding to HIV envelope glycoprotein. Mol Sys Des Eng, 4, 737-746, doi.org/10.1039/C8ME00080H (2019).

3) Also, several other journal names are not abbreviated in other references.

4) New ref 53 is also incomplete.

Please replace

Scharf, L. et al. Structural basis for germline antibody recognition of HIV-1 immunogens.572 Elife 5, doi:10.7554/eLife.13783 (2016).

with

Scharf, L. et al. Structural basis for germline antibody recognition of HIV-1 immunogens.572 eLife 5, e13783, doi:10.7554/eLife.13783 (2016).

5) In Table 2, please truncate values of B-factors (Å2) to integers and remove decimal points as they are not meaningful at these resolutions.

Reviewer #2 (Remarks to the Author):

I enjoyed reading the revised manuscript and feel the authors satisfactorily addressed the concerns

raised with the original draft. Figure 7 does a good job of contextualizing 3I14 with other stem-binding antibodies.

Reviewer #3 (Remarks to the Author):

The manuscript is improved. No major comments.

Minor:

Language referring to which protein is being bound is at time inaccurate. The Fab is binding HA and HA residues do not exist to be bound by Fabs. For example:

Lines 104-106 “H6 may also bind with improved affinity”
Should read “3I14 D93N may also bind H6 with improved affinity”

Lines 209-212. Discussion of the flexibility of Lys39. It is the Fab that accommodates the flexibility of this HA residue. The HA residue is not retaining a hydrogen bond with the Fab.

REVIEWERS' COMMENTS

Reviewer #1 (Remarks to the Author):

The authors have answered almost queries except and have updated the revised manuscript accordingly entitled “Targeting the Hemagglutinin Stem of Group 1 and 2 Influenza A Viruses: A Novel Solution from a VH3-30 Derived Antibody”. The revised manuscript is now suitable for publication.

Minor points to be addressed:

1) Page 12, line 4 from bottom of page. Please replace “germline encoded” with “germline-encoded”.

A: We thank the reviewer for their thorough editing of the manuscript and have updated to include a hyphen.

2) Page 20, Reference 32. Incomplete reference.
A: This comment is unclear: however, we have added an additional reference (now references 52-54).

This reference is incomplete as it does not have a volume (or issue) number.

Please replace

Alberto Cisneros, I., et al. . Role of antibody heavy and light chain interface residues in affinity maturation of binding to HIV envelope glycoprotein. Molecular Systems Design & Engineering, 737-746, doi:doi.org/10.1039/C8ME00080H (2019).

with

Alberto Cisneros, I., et al. Role of antibody heavy and light chain interface residues in affinity maturation of binding to HIV envelope glycoprotein. Mol Sys Des Eng, 4, 737-746, doi.org/10.1039/C8ME00080H (2019).

A: We thank the reviewer for the clarity and have updated the reference accordingly.

3) Also, several other journal names are not abbreviated in other references.

A: We have carefully gone through the references and updated accordingly.

4) New ref 53 is also incomplete.

Please replace

Scharf, L. et al. Structural basis for germline antibody recognition of HIV-1 immunogens.572

Elife 5, doi:10.7554/eLife.13783 (2016).

with

Scharf, L. et al. Structural basis for germline antibody recognition of HIV-1 immunogens.572 eLife 5, e13783, doi:10.7554/eLife.13783 (2016).

A: We have updated this reference accordingly.

5) In Table 2, please truncate values of B-factors (Å2) to integers and remove decimal points as they are not meaningful at these resolutions.

A: We have edited the table to remove decimal points.

Reviewer #2 (Remarks to the Author):

I enjoyed reading the revised manuscript and feel the authors satisfactorily addressed the concerns raised with the original draft. Figure 7 does a good job of contextualizing 3I14 with other stem-binding antibodies.

Reviewer #3 (Remarks to the Author):

The manuscript is improved. No major comments.

Minor:

Language referring to which protein is being bound is at time inaccurate. The Fab is binding HA and HA residues do not exist to be bound by Fabs. For example:

Q: Lines 104-106 "H6 may also bind with improved affinity"
Should read "3I14 D93N may also bind H6 with improved affinity"

A: We thank the reviewer and have updated the text to ensure accuracy.

Q: Lines 209-212. Discussion of the flexibility of Lys39. It is the Fab that accommodates the flexibility of this HA residue. The HA residue is not retaining a hydrogen bond with the Fab.

A: We thank the reviewer for pointing out this inaccuracy and have updated the text to read: "The ability for Lys39HA to adopt multiple rotamers and for 3I14 and 3I14D93N to retain a hydrogen bond interaction in either instance explains the similar binding affinities for each antibody towards group 2 HAs."
